# Supplementary material for: Chemical capture of diazo metabolites reveals biosynthetic hydrazone oxidation
Source: Nature. 2026 Feb 4;652(8109):517–25. doi: 10.1038/s41586-025-10079-x (PMC13061610; doi:10.1038/s41586-025-10079-x)
Supplement: Supplementary file 1 — Supplementary Methods, Tables 1–7, Figs. 1–46 and references. [file 41586_2025_10079_MOESM1_ESM.pdf]

nature portfolio

https://doi.org/10.1038/s41586-025-10079-x

Supplementary information

Chemical capture of diazo metabolites reveals biosynthetic hydrazone oxidation

In the format provided by the authors and unedited

Supporting Information

Synthesis of (*S*)-6-(3-((2-amino-2-carboxyethoxy)carbonyl)-1,9-dihydro-8*H*-dibenzo[*b*,*f*]pyrazolo[4,3-*d*]azocin-8-yl)-6-oxohexanoic acid & (*S*)-6-(3-((2-amino-2-carboxyethoxy)carbonyl)-1,8-dihydro-9*H*-dibenzo[*b*,*f*]pyrazolo[3,4-*d*]azocin-9-yl)-6-oxohexanoic acid (3 regioisomers)

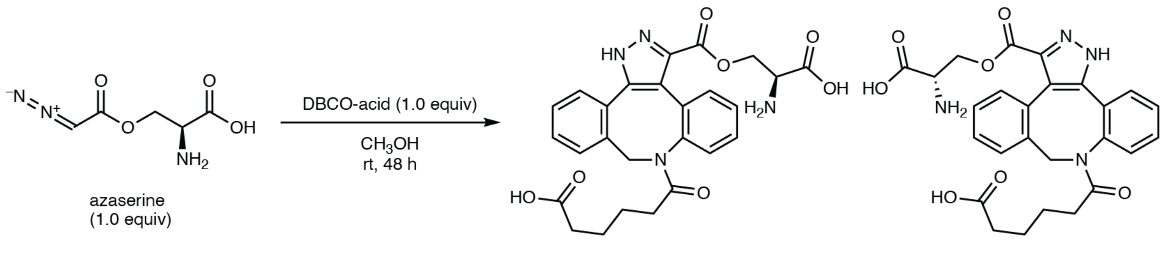

3 regioisomers

A suspension of azaserine (5.85 mg, 33.8 μmol, 1.0 equiv.) and DBCO-acid (11.3 mg, 33.8 μmol, 1.0 equiv.) in MeOH (1.35 mL) was stirred for 48 h, upon which the suspension dissolved completely to yield a colorless solution. The reaction mixture was directly purified by preparatory-HPLC (5 – 50% ACN in water, with 0.1% formic acid additive throughout, over 30 min) to yield an inseparable mixture of regioisomers of **1** (5.0 mg, 9.87 μmol, 29%) as a fluffy white powder after lyophilization.

^{1}H NMR (Supplementary Figure 26): (400 MHz, CD_3OD) δ 7.73 – 6.95 (m, 8H), 5.98 (d, *J* = 16.7 Hz, 0.6H), 5.66 (dd, *J* = 15.4, 9.9 Hz, 0.4H), 4.79 – 4.63 (m, 1.6H), 4.58 – 4.38 (m, 1.4H), 4.08 – 3.89 (m, 1H), 2.23 – 2.04 (m, 2H), 1.95 – 1.72 (m, 1.6H), 1.65 (dd, *J* = 15.8, 7.4 Hz, 0.4H), 1.45 – 1.25 (m, 4H).

^{13}C NMR (Supplementary Figure 27): (101 MHz, CD_3OD) δ 177.84, 177.55, 175.12, 174.63, 170.81, 170.53, 141.71, 141.23, 141.19, 136.15, 134.81, 134.51, 134.41, 133.24, 133.22, 132.95, 132.77, 132.05, 130.91, 130.88, 130.66, 130.56, 130.52, 130.38, 129.84, 129.62, 129.58, 129.06,

128.51, 128.45, 128.41, 128.24, 128.16, 65.03, 64.78, 55.30, 55.19, 53.40, 52.87, 34.87, 34.85, 34.74, 34.70, 34.66, 25.59, 25.54, 25.51, 25.47, 25.40.

HRMS (ESI+, *m/z*): $[M+H]^+$ calculated for $C_{26}H_{27}N_4O_7^+$, 507.1874; found 507.1876

Synthesis of methyl 4-diazo-3-oxobutanoate (S1)

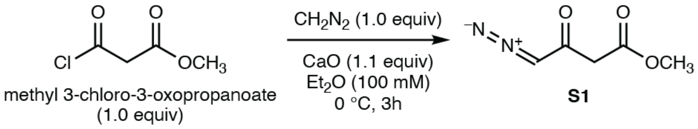

A freshly prepared and titrated solution of diazomethane in diethyl ether (0.25 M, 4.0 mL, 1.0 mmol, 1.0 equiv)[71] was added to a suspension of calcium oxide (61.7 mg, 1.0 mmol, 1.1 equiv) in anhydrous diethyl ether (5.0 mL) at 0 ºC.[72] The suspension was stirred at 0 ºC for 5 min, and methyl 3-chloro-3-oxopropanoate (0.107 mL, 1.0 mmol, 1.0 equiv) in anhydrous diethyl ether (0.5 mL) was added dropwise. The reaction mixture was stirred at 0 ºC for 3 h, allowed to warm to room temperature, filtered, and concentrated under reduced pressure (ambient water bath) to yield a crude light-yellow oil. Purification by flash-column chromatography (30 – 50% ethyl acetate in hexanes) afforded methyl 4-diazo-3-oxobutanoate (85.0 mg, 0.598 mmol, 60% yield) as a light-yellow oil.

^{1}H NMR (Supplementary Figure 28): (400 MHz, $CDCl_3$) δ 5.53 (s, 1H, N^-=N^+−C**H**−CO), 3.74 (s, 3H, −OC**H₃**), 3.36 (s, 2H, CO−C**H₂**−CO).

^{13}C NMR (Supplementary Figure 29): (101 MHz, $CDCl_3$) δ 186.07 (N^-=N^+−CH−**C**O), 167.85 (−**C**OOCH₃), 56.12 (N^-=N^+−**C**H−CO), 52.68 (−O**C**H₃), 46.87 (CO−**C**H₂−CO).

FTIR (neat), cm^{-1}: 3102 (m), 2956 (w), 2107 (s), 1739 (s), 1636 (s).

HRMS (ESI+, *m/z*): $[M+H–N_2]^+$ calculated for $C_5H_7O_3^+$, 115.0390; found 115.0387.

Synthesis of 6-(3-(3-methoxy-3-oxopropanoyl)-1,9-dihydro-8*H*-dibenzo[*b*,*f*]pyrazolo[4,3-*d*]azocin-8-yl)-6-oxohexanoic acid & 6-(3-(3-methoxy-3-oxopropanoyl)-1,8-dihydro-9*H*-dibenzo[*b*,*f*]pyrazolo[3,4-*d*]azocin-9-yl)-6-oxohexanoic acid (4-OMe regioisomers)

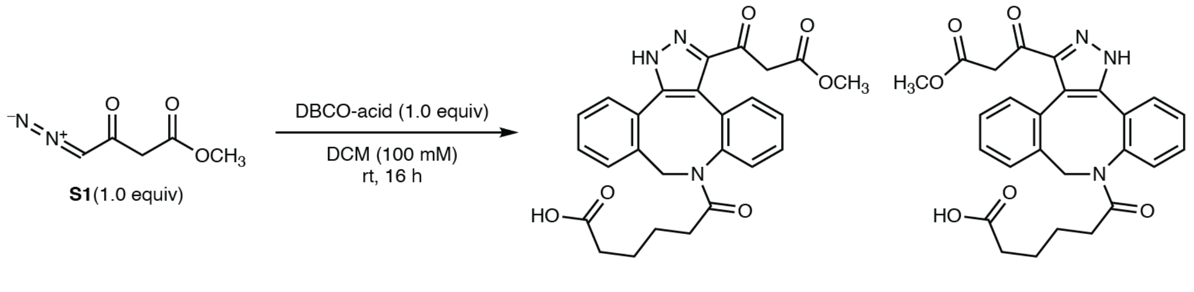

4-OMe regioisomers

DBCO-C_6-Acid (14.00 mg, 42.0 µmol, 1.0 equiv) was dissolved in DCM (420 µL) and **S1** (5.97 mg, 42.0 µmol, 1.0 equiv) dissolved in DCM (420 µL) was added. The colorless solution was stirred for 16 h, concentrated under reduced pressure, and purified by preparatory-HPLC (5 – 50% ACN in water, with 0.1% formic acid additive throughout, over 30 min) to yield an inseparable mixture of regioisomeric **4-OMe** in approximately a 2:1 ratio (16.7 mg, 35.1 µmol, 84%) as a fluffy white powder after lyophilization.

^{1}H NMR (Supplementary Figure 30): (400 MHz, $CDCl_3$) δ 7.53 – 7.47 (m, 1H), 7.47 – 7.32 (m, 4H), 7.30 – 7.15 (m, 5H), 7.13 – 7.08 (m, 1H), 6.94 – 6.88 (m, 1H), 5.82 (d, *J* = 17.0 Hz, 1H), 5.62 (d, *J* = 15.3 Hz, 0.5H), 4.44 (d, *J* = 15.4 Hz, 0.5H), 4.39 (d, *J* = 16.3 Hz, 1H), 4.19 (d, *J* = 17.1 Hz, 1H), 4.00 (d, *J* = 16.2 Hz, 1H), 3.84 (d, *J* = 9.5 Hz, 0.5H), 3.77 (d, *J* = 8.4 Hz, 0.5H), 3.74 (s, 3H), 3.70 (s, 1.5H), 2.31 – 2.11 (m, 3H), 1.93 – 1.79 (m, 3H), 1.76 – 1.69 1.72 (m, 1H), 1.58 – 1.20 (m, 7H).

^{13}C NMR (Supplementary Figure 31): (101 MHz, $CDCl_3$) δ 189.50, 185.77, 178.19, 177.24, 174.49, 173.03, 169.21, 167.78, 147.31, 146.04, 142.42, 141.18, 140.57, 139.65, 134.56, 134.33, 132.86, 132.20, 132.16, 132.08, 131.45, 131.10, 130.82, 130.65, 129.83, 129.62, 129.16, 129.11, 128.92, 128.63, 127.71, 126.91, 126.55, 126.13, 123.44, 117.25, 52.92, 52.64, 52.46, 52.18, 46.55, 45.97, 33.88, 33.49, 33.43, 24.36, 24.27, 24.23, 23.90.

HRMS (ESI+, *m/z*): $[M + H]^+$ calculated for $C_{26}H_{26}N_3O_6^+$, 476.1816; found 476.1824.

Synthesis of 6-(3-acetyl-1,9-dihydro-8*H*-dibenzo[*b*,*f*]pyrazolo[4,3-*d*]azocin-8-yl)-6-oxohexanoic acid (5 regioisomers)

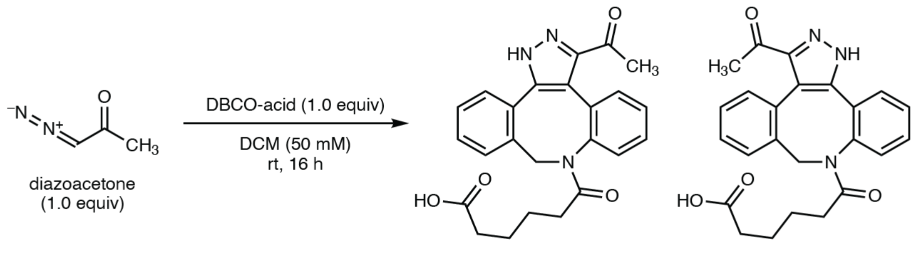

5 regioisomers

39.7 mg of DBCO-acid (0.12 mmol, 1.0 equiv) were added to 100 mg of diazoacetone (0.12 mmol, 1 equiv) (purchased from Enamine) in 2.4 mL of dichloromethane (DCM) in a capped vial. The reaction mixture was stirred overnight, and 0.5 mL of DCM were added the next morning to replace evaporated solvent. The mixture was concentrated under vacuum and purified using reversed phase chromatography on a Biotage Selekt using a 6 g C18 column with a 2% to 95% gradient of ACN in H_2O. Fractions were concentrated under reduced pressure and then lyophilized. The product was re-purified using a Dionex UltiMate HPG-3200Bx Semi-Preparative HPLC using a HypersilGold column with a 5% to 95% ACN (+ 0.1% formic acid) in H_2O (+ 0.1% formic acid) gradient. The fractions were concentrated under reduced pressure and lyophilized. NMR spectra were obtained using a Bruker AVANCE NEO 400B spectrometer (400 MHz, 100 MHz) and correspond to a 1:1 mixture of both pyrazole regioisomers. HRMS data were collected using a Thermo Orbitrap IQ-X Tribrid mass spectrometer coupled to a Horizon Vanquish UHPLC as described above.

^{1}H NMR (Supplementary Figure 32): (400 MHz, $CDCl_3$) δ 7.50 – 7.32 (m, 7H), 7.30 – 7.27 (m, 2H), 7.25 – 7.17 (m, 11H), 7.07 – 6.99 (m, 2H), 5.92 (d, *J* = 16.9 Hz, 1H), 5.77 (d, *J* = 15.5 Hz, 1H), 4.42 (d, *J* = 15.5 Hz, 1H), 4.26 (d, *J* = 17.0 Hz, 1H), 2.64 (s, 3H), 2.31 (s, 3H), 2.26 – 2.12 (m, 3H), 1.84 (m, 3H), 1.71 (m 1H), 1.53 – 1.22 (m, 4H).

^{13}C NMR (Supplementary Figure 33): (101 MHz, $CDCl_3$) δ 194.50, 190.58, 178.09, 177.44, 174.14, 173.27, 148.89, 145.76, 143.20, 140.45, 140.07, 139.78, 134.78, 134.49, 133.34, 132.52, 132.11, 131.99, 131.06, 130.76, 130.49, 129.84, 129.57, 129.27, 129.20, 128.63, 128.57, 128.46, 127.59, 127.11, 126.78, 126.72, 123.35, 117.42, 52.70, 52.26, 33.88, 33.66, 33.52, 28.63, 27.76, 24.27, 24.20, 23.93.

HRMS (ESI+, *m/z*): $[M + H]^+$ calculated for $C_{24}H_{24}N_3O_4^+$, 418.1761; found 418.1760.

Synthesis of methyl (*E*)-4-hydrazono-3-oxobutanoate (6-OMe)

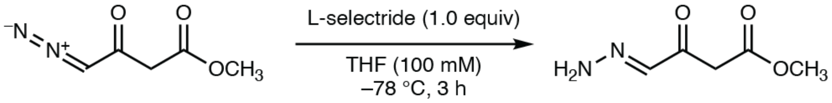

S1 (1.0 equiv) **6-OMe**

S1 (15.0 mg, 106 μmol, 1.0 equiv) was dissolved in tetrahydrofuran (THF) (1.06 mL) and cooled to –78 ºC. L-selectride in THF (1.0 M, 106 μL, 1.0 equiv) was added dropwise and the resultant canary-yellow solution was stirred at the same temperature for 3 h.[73] The reaction mixture was quenched at –78 ºC with MeOH (42.7 μL, 10 equiv), warmed to room temperature, and concentrated under reduced pressure. Purification by flash-column chromatography (0 – 5% MeOH in DCM) afforded methyl (*E*)-4-hydrazineylidene-3-oxobutanoate **(6-OMe)** (5.0 mg, 34.7 μmol, 33% yield) as a light-yellow film.

^{1}H NMR (Supplementary Figure 34): (400 MHz, CD_2Cl_2) δ 7.09 (s, 1H, H_2N−N=C**H**−CO), 6.49 (s, 2H, **H**$_2$N−N=), 3.72 (s, 2H, CO−C**H₂**−CO), 3.68 (s, 3H, −OC**H₃**).

^{13}C NMR (Supplementary Figure 35): (101 MHz, CD_2Cl_2) δ 192.14 (H_2N−N=CH−**C**O), 169.02 (−**C**OOCH$_3$), 137.67 (H_2N−N=**C**H−CO), 52.57 (−O**C**H$_3$), 43.99 (CO−**C**H$_2$−CO).

FTIR (neat), cm^{-1}: 3430 (m), 3308 (m), 3225 (m), 2954 (m), 1739 (s), 1664 (s), 1596 (s).

HRMS (ESI+, *m/z*): $[M+H]^+$ calculated for $C_5H_9N_2O_3^+$, 145.0608; found 145.0609.

NB: When dissolved in $CDCl_3$, the (*E*)-hydrazone was observed to isomerize to the (*Z*)-hydrazone.

Synthesis of *N*-6-hydroxylysine (8)

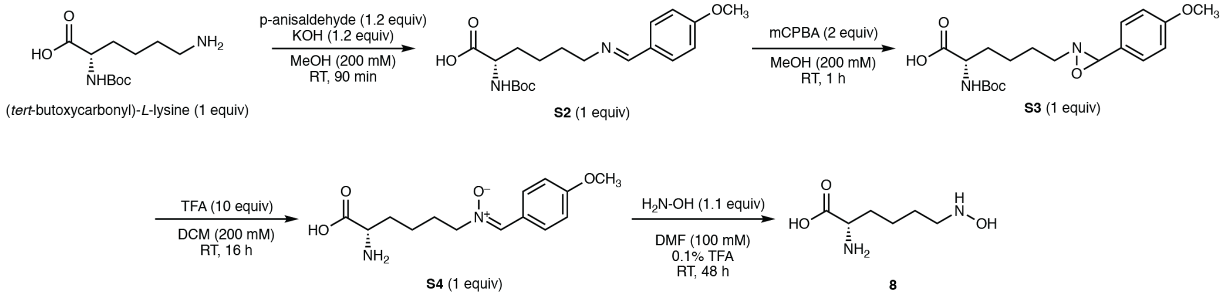

8 was synthesized according to a previously reported procedure.[20] NMR and HRMS data matched the previously reported values.

^{1}H NMR (Supplementary Figure 36): (400 MHz, D_2O) δ 3.73 (t, *J* = 6.1 Hz, 1H), 3.33 – 3.24 (m, 2H), 1.93-1.82 (m, 2H), 1.79-1.72 (m, 2H), 1.58 – 1.36 (m, 2H).

^{13}C NMR (Supplementary Figure 37): (101 MHz, D_2O) δ 172.03, 50.06.

Synthesis of hydrazinoacetic acid (10)

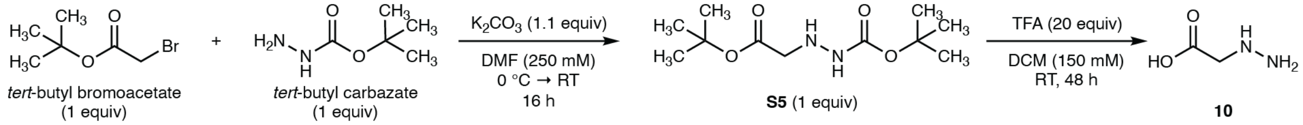

10 was synthesized according to a previously reported procedure.[20] NMR and HRMS data matched previously reported values.

^{1}H NMR (Supplementary Figure 38): (400 MHz, D_2O) δ 3.82 (s, 2H).

^{13}C NMR (Supplementary Figure 39): (101 MHz, D_2O) δ 172.03, 50.06.

Synthesis of Ethyl (*E*)-2-hydrazineylideneacetate (18)

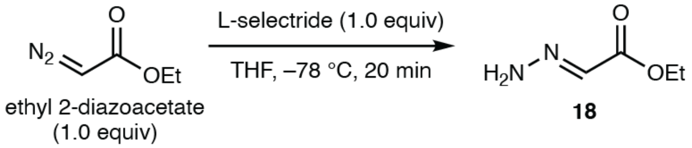

To a yellow solution of ethyl 2-diazoacetate (87 Wt% in DCM, 100 µL, 827 µmol, 1.0 equiv.) dissolved in THF (4.14 mL) at –78 °C was added 1.0 M L-selectride in THF (827 µL, 827 µmol, 1.0 equiv.) dropwise. The reaction was stirred at the same temperature for 20 min and quenched at –78 °C by the addition of a minimal amount of ethanol. The solution was allowed to warm up to room temperature and concentrated to yield a crude blood-orange oil. The crude oil was purified by flash column chromatography (50% ethyl acetate in hexanes) to yield **18** (48.0 mg, 413 µmol, 50%) as a colorless film.

^{1}H NMR (Supplementary Figure 40): (400 MHz, CD_2Cl_2) δ 7.02 (s, 1H, $H_2N{-}N{=}C\mathbf{H}{-}CO$), 6.38 (s, 2H, $\mathbf{H}_2N{-}N{=}$), 4.21 (q, *J* = 7.1 Hz, 2H, $-OC\mathbf{H_2}CH_3$), 1.29 (t, *J* = 7.2 Hz, 3H, $-OCH_2C\mathbf{H_3}$).

^{13}C NMR (Supplementary Figure 41): (101 MHz, CD_2Cl_2) δ 164.46 ($H_2N{-}N{=}CH{-}\mathbf{C}O$), 130.02 ($H_2N{-}N{=}\mathbf{C}H{-}CO$), 61.12 ($-O\mathbf{C}H_2CH_3$), 14.60 ($-OCH_2\mathbf{C}H_3$).

HRMS (ESI+, *m/z*): $[M+H]^+$ calculated for $C_4H_9N_2O_2^+$, 117.0659; found 117.0657.

Synthesis of (*E*)-2-hydrazineylidene-*N*,*N*-dimethylacetamide (19)

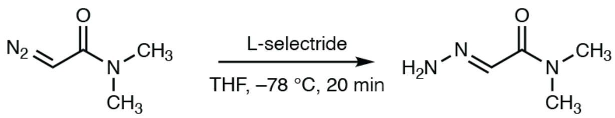

2-diazo-*N*,*N*-dimethylacetamide (1.0 equiv) **19**

To a yellow solution of 2-diazo-*N,N*-dimethylacetamide[74] (68 mg, 601 µmol, 1 equiv.) dissolved in THF (3.0 mL) at –78 °C was added 1.0 M L-selectride in THF (601 µL, 601 µmol, 1.0 equiv.) dropwise. The reaction was stirred at the same temperature for 20 min and quenched at –78 °C by the addition of a minimal amount of ethanol. The solution was allowed to warm up to room temperature and concentrated to yield a crude orange oil. The crude oil was purified by flash column chromatography (0 – 5% MeOH in DCM) to yield **19** (38.2 mg, 332 µmol, 55%) as a white film.

^{1}H NMR (Supplementary Figure 42): (400 MHz, CD_2Cl_2) δ 7.33 (s, 1H, $H_2N-N=C\mathbf{H}-CO$), 5.94 (s, 2H, $\mathbf{H}_2N-N=$), 3.14 (s, 3H, $-N(C\mathbf{H_3})_2$), 2.95 (s, 3H, $-N(C\mathbf{H_3})_2$).

^{13}C NMR (Supplementary Figure 43): (101 MHz, CD_2Cl_2) δ 164.56 ($H_2N-N=CH-\mathbf{C}O$), 164.37* ($H_2N-N=CH-\mathbf{C}O$), 134.46 ($H_2N-N=\mathbf{C}H-CO$), 120.74* ($H_2N-N=\mathbf{C}H-CO$), 38.11 ($-N(\mathbf{C}H_3)_2$), 37.54* ($-N(\mathbf{C}H_3)_2$), 36.20 ($-N(\mathbf{C}H_3)_2$), 35.08* ($-N(\mathbf{C}H_3)_2$).

NB: The (*E*)-hydrazone was observed to isomerize (~50% conversion) to the (*Z*)-hydrazone over the timespan of the ^{13}C NMR experiment in CD_2Cl_2. Therefore, eight ^{13}C peaks, rather than the expected four, were observed. Peaks corresponding to the (Z)-hydrazone are labeled with an asterisk.

HRMS (ESI+, *m/z*): $[M+H]^+$ calculated for $C_4H_{10}N_3O^+$, 116.0818; found 116.0818.

Synthesis of 6-oxo-6-(3-phenyl-1,9-dihydro-8*H*-dibenzo[*b*,*f*]pyrazolo[4,3-*d*]azocin-8-yl)hexanoic acid & 6-oxo-6-(3-phenyl-1,8-dihydro-9*H*-dibenzo[*b*,*f*]pyrazolo[3,4-*d*]azocin-9-yl)hexanoic acid (20 regioisomers)

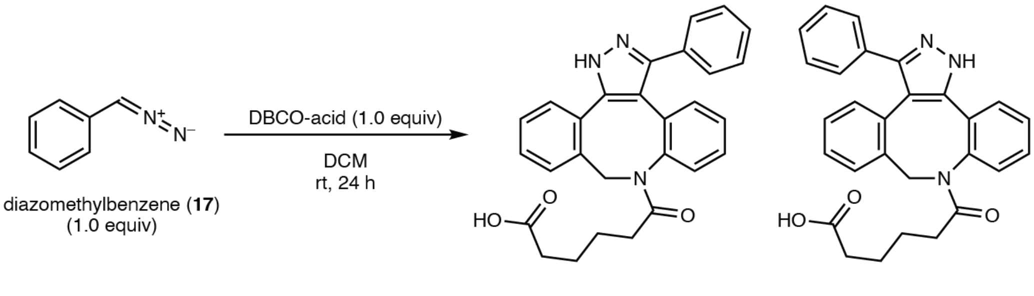

20 regioisomers

A peach-colored solution of diazomethylbenzene (19.7 mg, 167 µmol, 1.33 equiv.) in DCM-diethyl ether-pentane[75] (1:9:10 ratio, 3.34 mL) was added to DBCO-acid (41.6 mg, 125 µmol, 1.0 equiv.) in DCM (333 µL) and stirred for 24 h. The reaction mixture was concentrated and purified by flash column chromatography (0 – 5% MeOH in DCM) to yield an inseparable mixture of regioisomeric **20** (12.0 mg, 26.6 µmol, 16%) as a fluffy white powder after lyophilization.

^{1}H NMR (Supplementary Figure 44): (400 MHz, $CDCl_3$) δ 7.58 – 7.27 (m, 8.6H), 7.25 – 7.07 (m, 3.4H), 7.00 (dtd, *J* = 15.1, 7.5, 1.4 Hz, 0.6H), 6.79 (dd, *J* = 7.7, 1.4 Hz, 0.4H), 6.00 (d, *J* = 16.3 Hz, 0.6H), 5.93 (d, *J* = 15.6 Hz, 0.4H), 4.51 – 4.35 (m, 1H), 2.17 – 1.96 (m, 2.6H), 1.91 – 1.78 (m, 1H), 1.54 – 1.17 (m, 4.4H).

^{13}C NMR (Supplementary Figure 45): (101 MHz, $CDCl_3$) δ 177.69, 177.18, 173.12, 172.80, 147.85, 147.06, 143.18, 142.73, 140.94, 140.71, 134.75, 134.65, 133.51, 133.14, 132.86, 131.95, 131.90, 131.50, 130.44, 130.25, 130.12, 129.64, 129.42, 129.38, 129.33, 129.19, 128.94, 128.89, 128.81, 128.76, 128.72, 128.50, 128.40, 127.90, 127.50, 127.47, 127.11, 118.21, 115.16, 53.03, 52.88, 34.14, 33.94, 33.92, 33.75, 24.51, 24.40, 24.26.

HRMS (ESI+, *m/z*): $[M+H]^+$ calculated for $C_{28}H_{26}N_3O_3^+$, 452.1969; found 452.1974.

Supplementary Table 1: Primers used in this study.

**Primers**	**Sequence (5’-3’)**	**Description**
DobA_ninae_NcoI_HindIII_F	ctttaagaaggagatatacATGACCGCGGTCCTGC	HindIII
DobA_ninae_NcoI_HindIII_R	ggtgctcgagtgcggccgcaCGTTGCTCCTGGGACAGC	NcoI
DobB_ninae_NdeI_HindIII_F	ggtgccgcgcggcagccatATGGGATGGGCTGAGCTGG	NdeI
DobB_ninae_NdeI_HindIII_R	tgctcgagtgcggccgcaTCATTCGACGGCGCCC	HindIII
DobC_ninae_NdeI_HindIII_F	ggtgccgcgcggcagccatATGGATTCGGTGATCAACC	NdeI
DobC_ninae_NdeI_HindIII_R	tgctcgagtgcggccgcaTCAGGCATGTGTTCTTCC	HindIII
DobE_ninae_NdeI_HindIII_F	ggtgccgcgcggcagccatATGATCATCAATCGTTTCGACAC	NdeI
DobE_ninae_NdeI_HindIII_R	tgctcgagtgcggccgcaTCAAGCGCTCCCCAC	HindIII
DobF_ninae_NdeI_HindIII_F	ggtgccgcgcggcagccatATGACGAACA	NdeI

	ACACCGTCGC	
DobF_ninae_NdeI_HindIII_R	tgctcgagtgcggccgcaTCACGCGGACTCATCTC	HindIII
DobG_ninae_NdeI_HindIII_F	ggtgccgcgcggcagccatATGAGCCGGAGCTCGC	NdeI
DobG_ninae_NdeI_HindIII_R	tgctcgagtgcggccgcaTCATCGCTCTACGCTCCGG	HindIII
DobM_ninae_NdeI_HindIII_F	ggtgccgcgcggcagccatATGAGCTTCGATGCGGGC	NdeI
DobM_ninae_NdeI_HindIII_R	tgctcgagtgcggccgcaTCATTTCGCCCGCCCC	HindIII
DobQ_ninae_NdeI_HindIII_F	ggtgccgcgcggcagccatATGCCTGATCTGGAAAAGGCC	NdeI
DobQ_ninae_NdeI_HindIII_R	tgctcgagtgcggccgcaTCAGACCGCACTTTCGGAG	HindIII
Dob2_ninae_NdeI_HindIII_F	ggtgccgcgcggcagccatATGAGTGCGTTCCTGTCG	NdeI
Dob2_ninae_NdeI_HindIII_R	tgctcgagtgcggccgcaCTATCCCTCCATTTCTCTCCGG	HindIII
Dob3_ninae_NdeI_HindIII_F	ggtgccgcgcggcagccatATGACATTGCC	NdeI

	GCAATATCC	
Dob3_ninae_NdeI_HindIII_R	tgctcgagtgcggccgcaTCACTGTCCCTCGATCGTC	HindIII
Dob3_E137A_F	ggtgtggtactg**cgc**gtcgagcatcgc	ctc → cgc
Dob3_E137A_R	gcgatgctcgac**gcg**cagtaccacacc	gag → gcg
Dob3_E101A_F	tcaccacgtgctg**cgc**ggtgtccatgatg	ctc → cgc
Dob3_E101A_R	catcatggacacc**gcg**cagcacgtggtga	gag → gcg
Dob3_H140A_F	gagatgcatgagggt**ggc**gtactgctcgtcgagc	gtg → ggc
Dob3_H140A_R	gctcgacgagcagtac**gcc**accctcatgcatctc	cac → gcc
Dob3_H225A_F	ctcgtcgcggtt**ggc**catggtcgcggtg	gtg → ggc
Dob3_H225A_R	caccgcgaccatg**gcc**aaccgcgacgag	cac → gcc
Dob3_E198A_F	gttgatcgagat**cgc**ggcgaccgtggc	ctc → cgc
Dob3_E198A_R	gccacggtcgcc**gcg**atctcgatcaac	gag → gcg
Dob3_E229A_F	ggagtggcagta**cgc**gtcgcggttgtg	ctc → cgc
Dob3_E229A_R	cacaaccgcgac**gcg**tactgccactcc	gag → gcg
Dob3_H232A_F	cggcgatcgagga**ggc**gcagtactcgtcgc	gtg → ggc
Dob3_H232A_R	gcgacgagtactgc**gcc**tcctcgatcgccg	cac → gcc

Supplementary Table 2: Vectors used in this study

Vector	Description	Source
pET-28a(+)	Protein expression vector	Invitrogen
pDualP	Dual inducible BAC for whole-cluster expression	Terra Bioforge

Supplementary Table 3: Strains used in this study.

Strain	Description	Source
Glycomyces harbinensis ATCC 43155	Azaserine producer	ATCC
Streptomyces coelicolor M1152	Heterologous host for *dob* gene cluster expression	Lab stock
Escherichia coli Top10	Maintenance of protein expression vectors	Invitrogen
Escherichia coli BL21(DE3)	Protein expression	Invitrogen
Escherichia coli BAP1	Expression of phosphopantetheinylated proteins	Lab stock
Escherichia coli BacOpt2.0	Dh10B derivative – maintenance of *dob*-pDualP and *dob*-pDualP ΔDob3	Terra Bioforge
Escherichia coli ET12567/pUZ8002	Methylation deficient donor for *dob*-pDualP and *dob*-pDualP ΔDob3 conjugation	Lab Stock
Nocardia ninae NBRC 108245	Production of **1** and **2**	DSMZ
Nocardia tenerifensis DSM 44704	Production of **1** and **2**	DSMZ
Streptomyces coelicolor dob-pDualP	Cluster confirmation	This study
Streptomyces coelicolor dob-pDualP Δ*dob3*	In vivo investigation	This study

Supplementary Table 4: Protein sequences used in genome mining

Protein	Pfam	Accession ID (NCBI)
AzaA	03417	WP_218125324.1
AzaB	13302	WP_091038445.1
AzaC	00501	WP_218125325.1
AzaE	07883, 09334	WP_091038156.1
AzaF	01266	WP_091038158.1
AzaG	13434	WP_091038161.1
AzaM	02770	WP_091038175.1

Supplementary Table 5: Annotations of proteins in the *dob* biosynthetic gene cluster in *Nocardia ninae*.

Name	Size (aa)	Annotation	Homolog origin	Proposed function	Accession ID (NCBI)	ID (% aa)	Query coverage (%)
DobA	350	C45 peptidase	*Nocardia* sp. CS682	hydrazone-formation	WP_135232797.1	96.0	100
DobB	204	GCN5 *N*-acetyl transferase	*Nocardia* sp. XZ_19_369	hydrazone-formation	WP_194834407.1	99.0	100
DobC	495	AMP-dependent synthetase	*Nocardia* sp. CS682	hydrazone-formation	WP_135232795.1	98.0	100
DobE	671	Methionine tRNA ligase/cupin domain	*Nocardia* sp. CS682	hydrazone-formation	WP_206055239.1	97.8	100
DobF	358	FAD-binding oxidoreductase	*Nocardia* sp. XZ_19_369	hydrazone-formation	WP_194834410.1	96.1	100
DobG	439	L-lysine 6-monooxygenase	*Nocardia* sp. CS682	hydrazone-formation	WP_135232793.1	98.4	100
DobM	391	Acyl-CoA dehydrogenase	*Nocardia* sp. CS682	hydrazone-formation	WP_135232798.1	97.4	100
DobQ	86	Acyl carrier protein	*Nocardia suismassiliense*	hydrazone-formation	WP_107658748.1	98.8	100
Dob1	342	ParB transcriptional regulator	*Nocardia* sp. CS682	regulation	WP_135232792.1	98.3	100
Dob2	1239	Polyketide synthase	*Nocardia* sp. XZ_19_369	PKS	WP_194834404.1	95.8	100
Dob3	322	Ferritin-like diiron oxidase or oxygenase	*Nocardia* sp. CS682	Diazo formation	WP_135232800.1	98.8	100
Dob4	405	Major facilitator superfamily 1	*Nocardia suismassiliense*	Export/resistance	WP_107658754.1	97.8	100

Supplementary Table 6: Organisms containing *dob* biosynthetic gene clusters.

Organism	Description	Accession	Source database
Actinobacteria sp. 051321	Soil microbe	IMG 2931719839	IMG JGI
Actinomadura sp. J1-007	Industrial maduramicin producer	IMG 3003019599	IMG JGI
Nocardia ninae NBRC 108245	Human pathogen	NZ_BJXA00000000.1	NCBI
Nocardia tenerifensis DSM 44704	Animal pathogen	NZ_QJKF01000002.1	NCBI
Nocardia pseudobrasiliensis DSM 44290	Human pathogen	NZ_QQBC01000001.1	NCBI
Nocardia colli CICC11023	Human pathogen	NZ_VXLC01000004.1	NCBI
Nocardia suismassiliense S-137	Boar gut microbe	NZ_LT985361.1	NCBI
Nocardia sp. CS682	Soil microbe	CP029710.1	NCBI
Nocardia sp. NPDC 6044	Soil microbe	NZ_JBIAID010000001.1	NCBI
Nocardia sp. NPDC 50175	Soil microbe	NZ_JBITJI010000016.1	NCBI
Nocardia sp. NPDC 51321	Soil microbe	NZ_JBITEM010000001.1	NCBI
Nocardia sp. NPDC 51756	Soil microbe	NZ_JBFAYV010000001.1	NCBI
Nocardia sp. NPDC 57030	Soil microbe	NZ_JBHUSN010000373.1	NCBI
Nocardia sp. NPDC 60255	Soil microbe	NZ_JBHXAU010000066.1	NCBI
Nocardia sp. NPDC 52316	Soil microbe	NZ_JBIUBB010000001.1	NCBI
Nocardia sp. NPDC 46473	Soil microbe	NZ_JBEYZJ010000003.1	NCBI
Halostreptopolyspora alba YIM 96095	Soil microbe	IMG 2861794813	IMG JGI

Supplementary Table 7: Protein sequences for queries used in previous genome mining efforts

Reference	Protein	NCBI Accession Number
55	Lom29	AHZ61863.1
	Lom30	AHZ61864.1
	Lom32	AHZ61866.1
	Lom33	AHZ61867.1
	Lom34	AHZ61868.1
	Lom35	AHZ61869.1
12	CreD	ALA99201.1
	CreE	ALA99202.1
	CreM	ALA99210.1
14	AzpL	BCN13448.1
20	AzaD	WP_091038154.1
	AzaH	WP_091038164.1
	AzaI	WP_091038167.1
	AzaL	WP_143014975.1
	AzaN	WP_091038177.1
	AzaP	WP_091038183.1

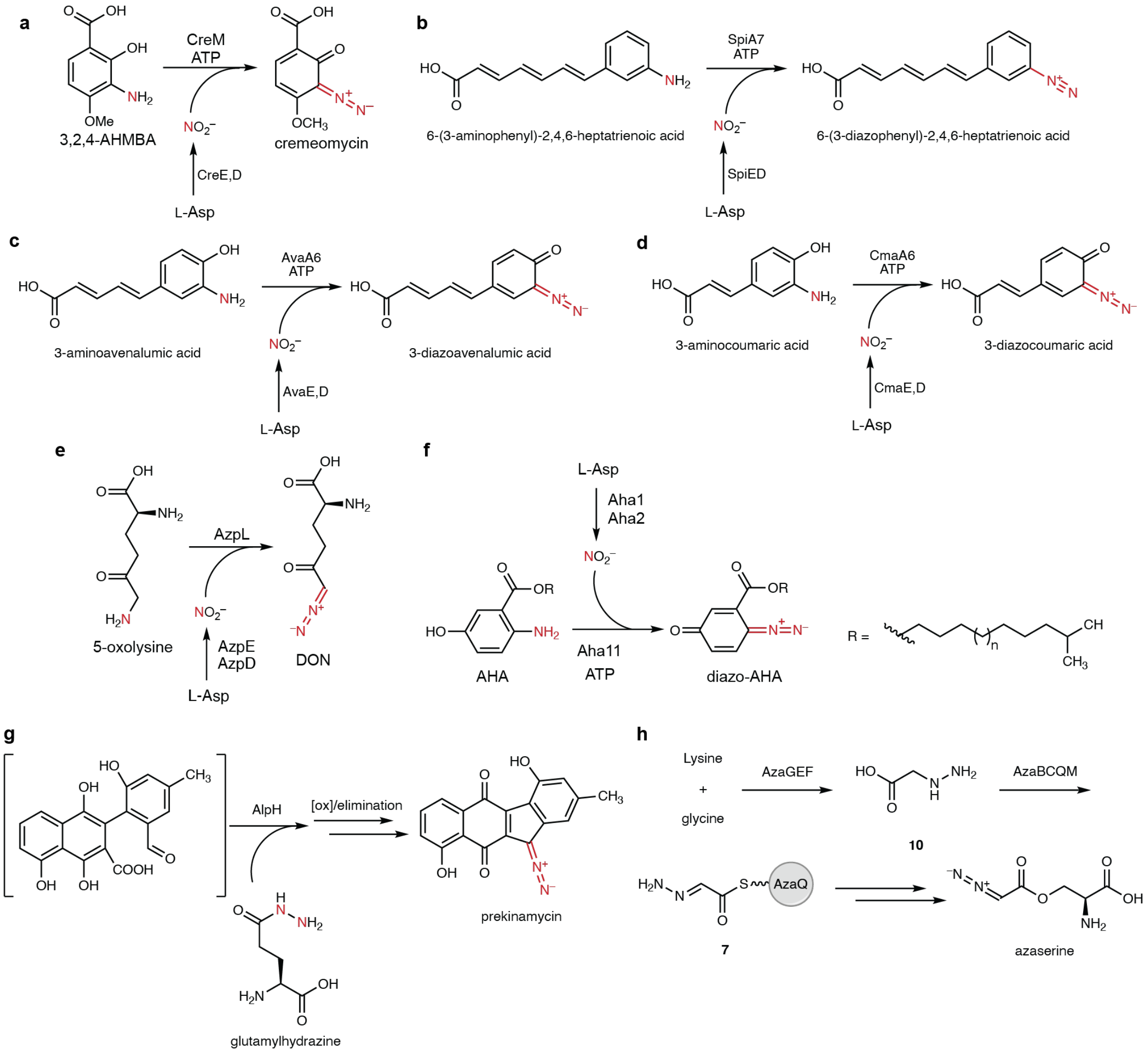

Supplementary Figure 1: Characterized and putative diazo biosynthetic enzymes. The first diazo-containing intermediate or product in each pathway is shown. **a)** CreM catalyzes formation of the diazo functionality in cremeomycin biosynthesis. 3,2,4-AHMBA = 3-amino-2-hydroxy-4-methoxybenzoic acid.[12,6] **b)** SpiA7 catalyzes diazo formation in spinamycin biosynthesis.[17] **c)** AvaA6 catalyzes 3-diazoavenalumic acid formation in avenalumic acid biosynthesis.[16] **d)** CmaA6 catalyzes 3-diazocoumaric acid formation in *p*-coumaric acid biosynthesis.[18] **e)** AzpL catalyzes DON formation in alazopeptin biosynthesis.[14] **f)** Aha11 catalyzes diazo formation in tasikamide biosynthesis. AHA = alkyl 5-hydroxylanthranilate.[15] **g)** AlpH catalyzes condensation of an acyl hydrazide with a carbonyl in kinamycin biosynthesis.[19] **h)** AzaGEF catalyzes conversion of L-lysine and glycine to **10** and subsequent transformations by AzaBCQM yields **7-AzaQ**.[20–22] The enzyme responsible for oxidation of the hydrazone to the diazo has not yet been elucidated.

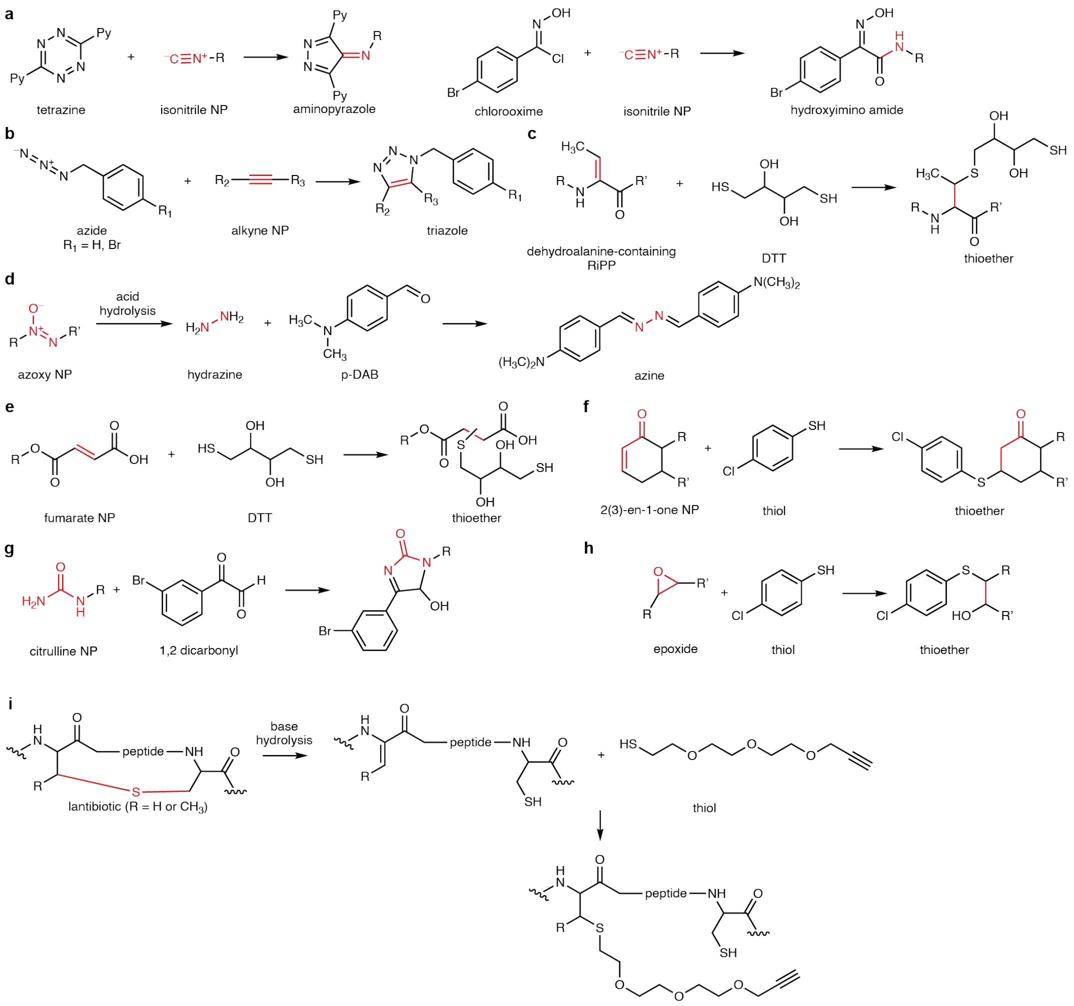

Supplementary Figure 2: Chemical trapping facilitates identification of natural products. **a)** Tetrazine[28] and chlorooxime[27] probes identify isonitrile-containing natural products. **b)** Azide probes undergo 1,3-dipolar cycloadditions to identify alkyne-containing natural products.[24,25] **c)** Dithiothreitol (DTT) undergoes 1,4-nucleophilic additions to identify dehydroalanine-containing RiPPs.[2,23] **d)** Acid hydrolysis of azoxy natural products yields hydrazine which can be derivatized by para-dimethylamino benzaldehyde (p-DAB) to identify azoxy natural products.[76] **e)** DTT undergoes 1,4-nucleophilic additions to identify fumarate-containing natural products.[77] **f)** Thiol probes undergo 1,4-nucleophilic additions to identify 2(3)-en-1-one-containing natural products.[78] **g)** 1,2-dicarbonyl probes identify citrulline-containing natural products.[79] **h)** Thiol probes identify epoxide-containing natural products.[80] **i)** Base hydrolysis of lantibiotics yields dehydrated amino acids which undergo 1,4-nucleophilic additions with thiol probes to introduce an alkyne functionality. A subsequent click reaction with an azide enables enrichment of the derivatized natural product.[81]

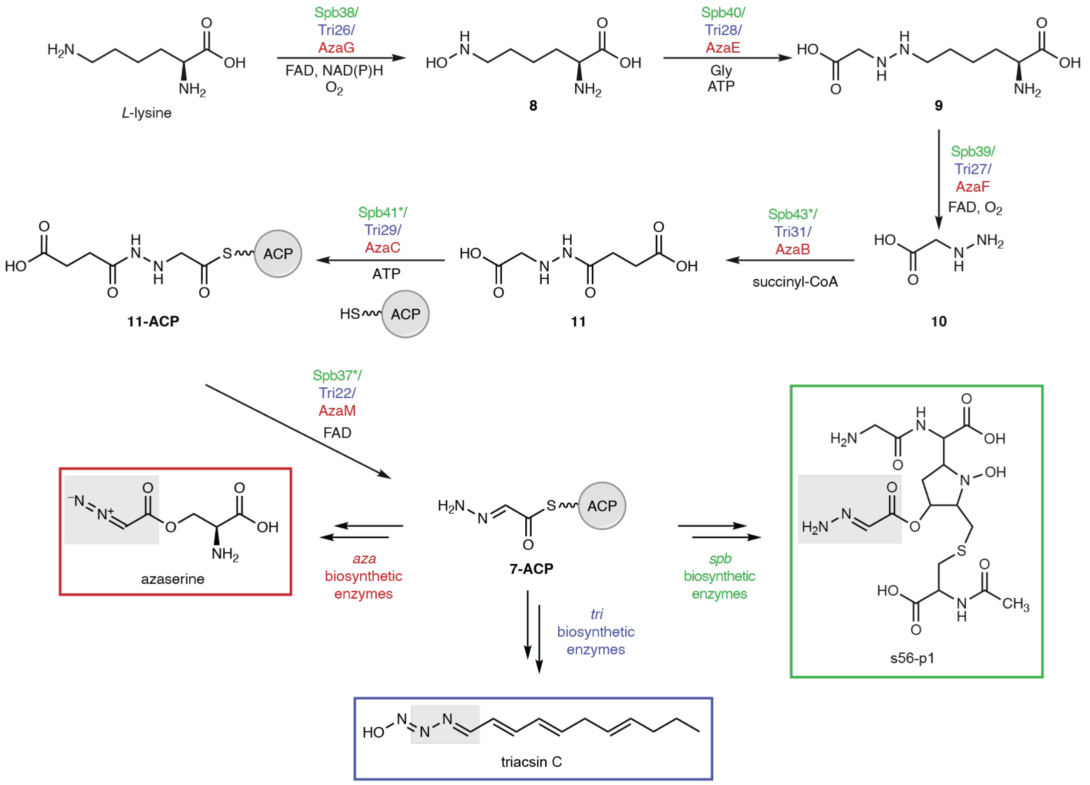

Supplementary Figure 3: Previously reported hydrazone biosynthetic pathways. Atoms highlighted in gray boxes originate from **10**. * = enzymes were identified bioinformatically and have not been biochemically characterized.

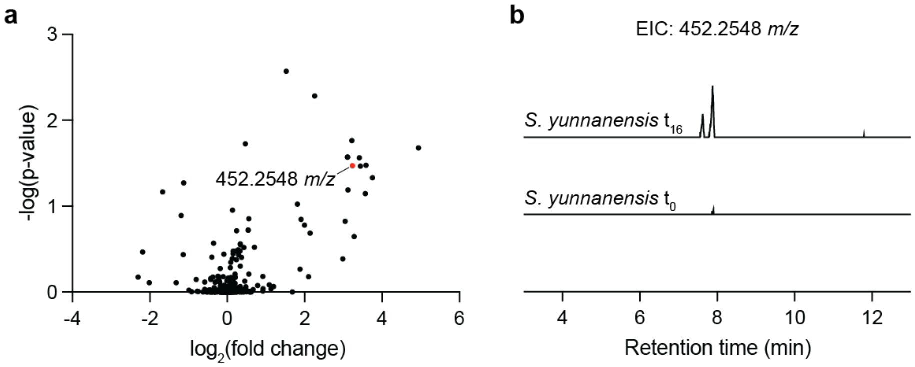

Supplementary Figure 4: Reactivity-guided comparative metabolomics identifies a putative diazo-containing natural product from *S. yunnanensis*. **a)** Volcano plot for comparative metabolomics of *S. yunnanensis* spent medium treated with DBCO-acid at t_{16} vs $t_{0.}$ P-values are calculated by a one-way ANOVA model with Tukey as post-hoc test. **b)** EICs of *m/z* = 452.2548 ± 5 ppm from DBCO-acid treat spent medium at t = 16 h and t = 0 h. Low abundance of this metabolite meant the more polar (lower retention time) regioisomer fell below the threshold of detection for the volcano plot, resulting in only a single detected mass feature. Manual inspection of the EICs from highly differentially produced mass features in the volcano plot (log_2(fold change) > 2) led to the observation of two peaks for *m/z* = 452.2548 ± 5 ppm, consistent with a DBCO-acid derivatized metabolite. This hit was de-prioritized due to its low abundance compared to that of the hit from *N. ninae*.

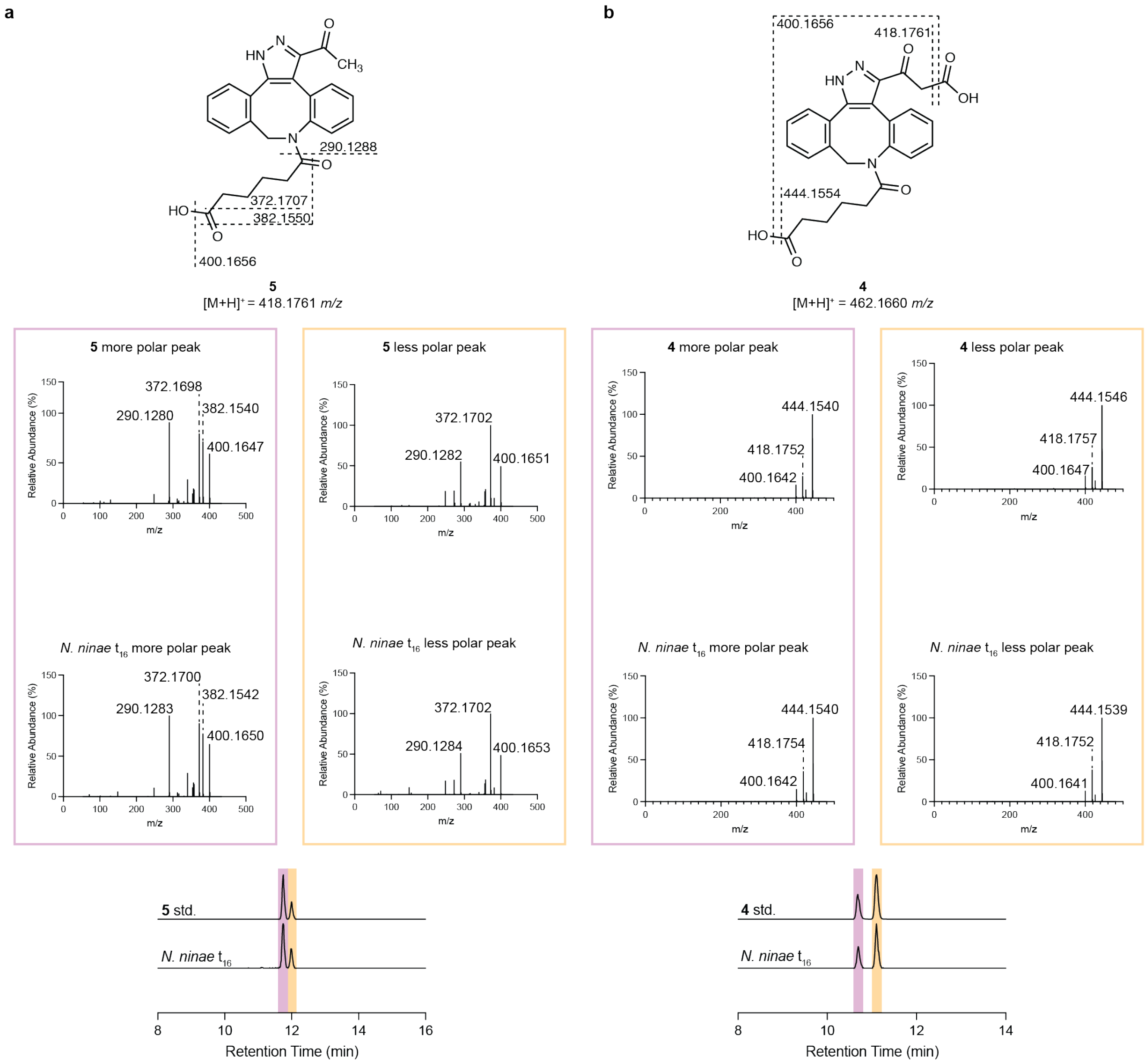

Supplementary Figure 5: MS/MS spectra of both regioisomers of synthetic **5** and **4**, and comparison with derivatized spent media from *N. ninae*. **a)** Fragmentation pattern of DBCO-acid derivatized *N. ninae* spent media matches that of synthetic **5**. Extracted ion chromatogram (EIC) (*m/z* = 418.1761 ± 5 ppm) of the DBCO-acid derivatized *N. ninae* spent media compared to a synthetic standard. **b)** Fragmentation pattern of DBCO-acid derivatized *N. ninae* spent media matches that of synthetic **4**. **4** was prepared through incubation of **4-OMe** with PLE. EIC (*m/z* = 462.1660 ± 5 ppm) of the DBCO-acid derivatized *N. ninae* spent media compared to a synthetic standard.

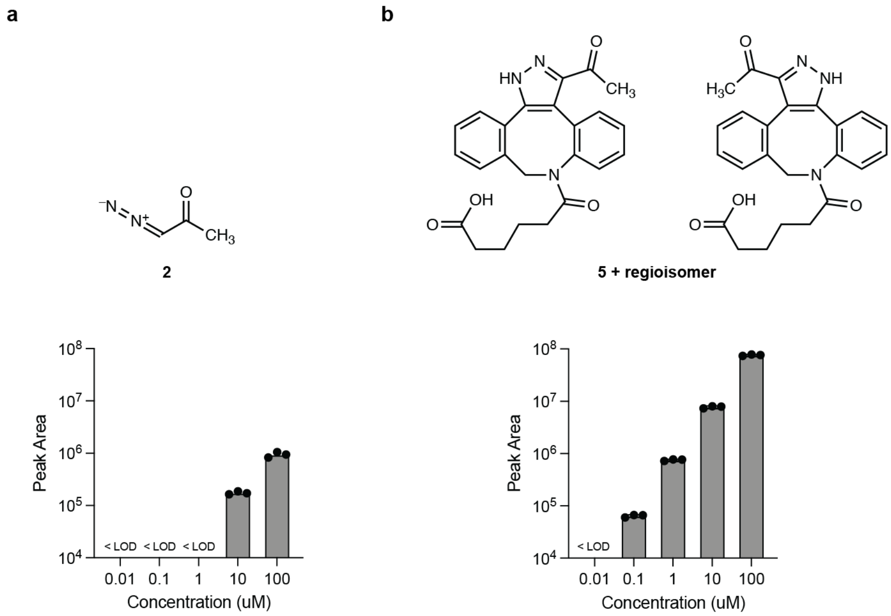

Supplementary Figure 6: DBCO-acid derivatization improves LC–MS sensitivity for detection of **2**. **a)** LC-MS peak areas of different starting concentrations of **2** in 10% ACN, 10% MeOH, and 80% H_2O. **b)** LC-MS peak areas of different starting concentrations of **5** in 10% ACN, 10% MeOH, and 80% H_2O. Peak areas were calculated as the sum of the regioisomer peaks. Error bars indicate mean ± standard deviation. Experiments were run in biological triplicates.

a

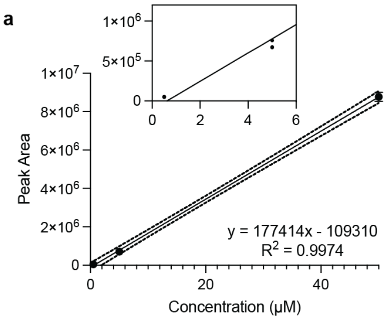

b

Area	Calculated concentration (μM)
225444	2
401394	3
302643	2
442382	3
average	3

c

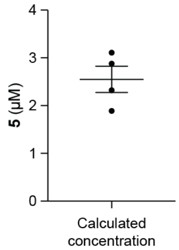

Supplementary Figure 7: Approximation of **5** in *N. ninae* spent medium. **a)** Standard curve of **5** at 500 nM, 5 μM, 50 μM in GYM medium. Peak areas from DBCO-derivatized *N. ninae* spent medium are highlighted in blue. Standards were run in biological triplicates. The dashed line indicates the 95% CI. Error bars indicate the SEM. **b)** Concentrations calculated from raw peak areas of *N. ninae* spent medium. *N. ninae* samples were run in biological quadruplicates. **c)** Concentration of **5** in *N. ninae* supernatants. The mean and SEM are shown.

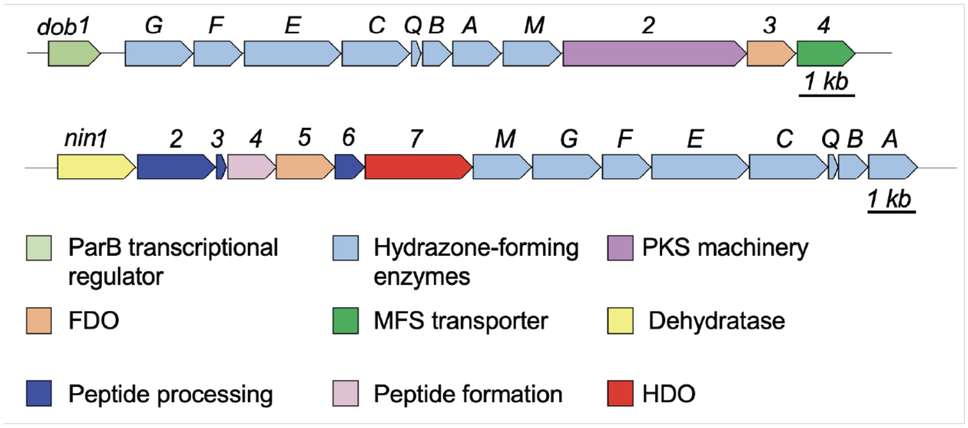

Supplementary Figure 8: Genome-mining for hydrazone-forming enzymes identified the *dob* and *nin* biosynthetic gene clusters in *N. ninae*. The PKS gene encoded in the *dob* gene cluster suggests *dob* is responsible for production of **1** and **2**.

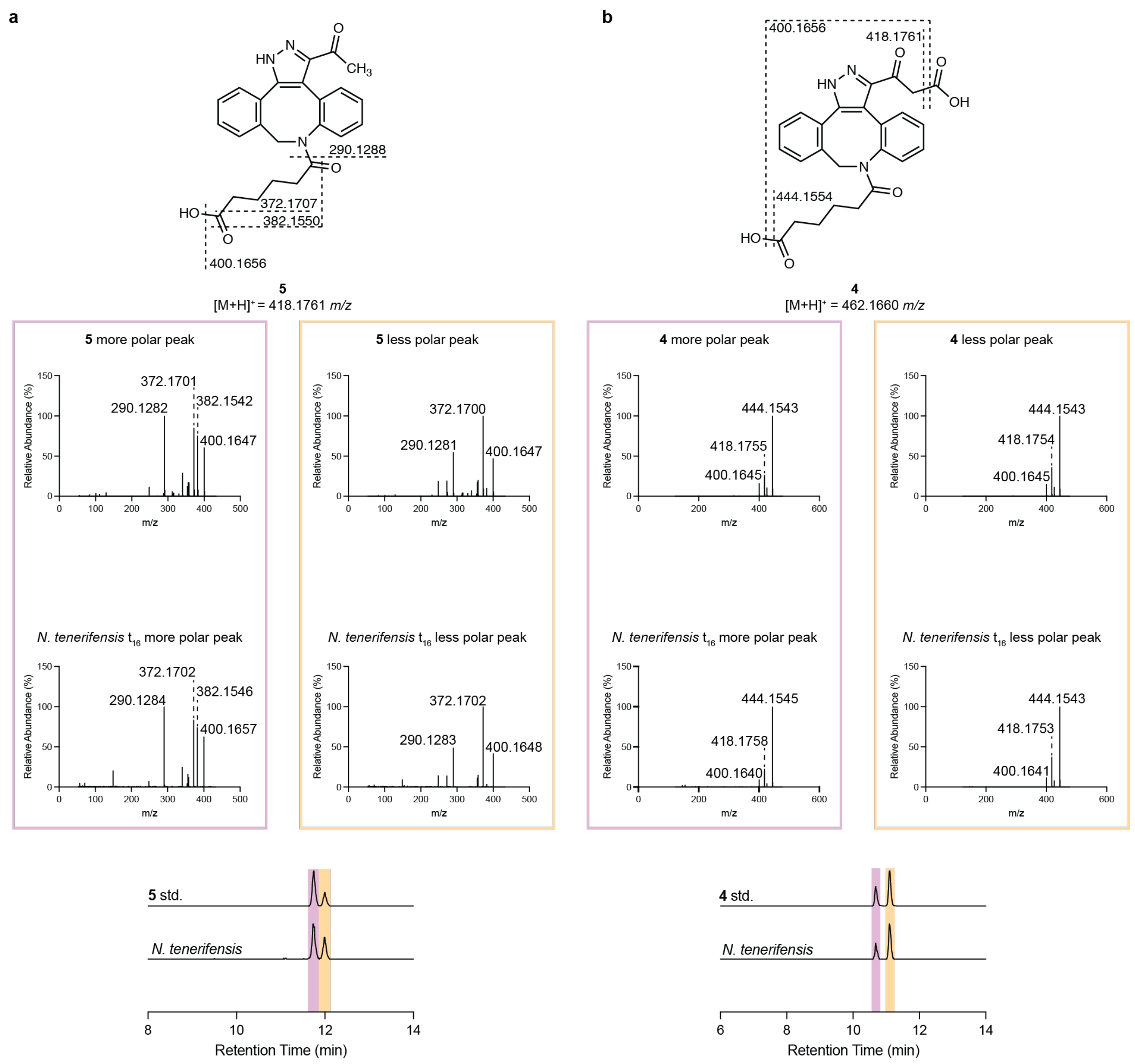

Supplementary Figure 9: MS/MS spectra of both regioisomers of synthetic **5** and **4**, and comparison with derivatized spent media from *N. tenerifensis*. **a)** Fragmentation pattern of DBCO-acid derivatized *N. tenerifensis* spent media matches that of synthetic **5**. EIC (*m/z* = 418.1761 ± 5 ppm) of the DBCO-acid derivatized *N. tenerifensis* spent media compared to a synthetic standard. **b)** Fragmentation pattern of DBCO-acid derivatized *N. tenerifensis* spent media matches that of synthetic **4**. **4** was prepared through incubation of **4-OMe** with PLE. EIC (*m/z* = 462.1660 ± 5 ppm) of the DBCO-acid derivatized *N. tenerifensis* spent media compared to a synthetic standard.

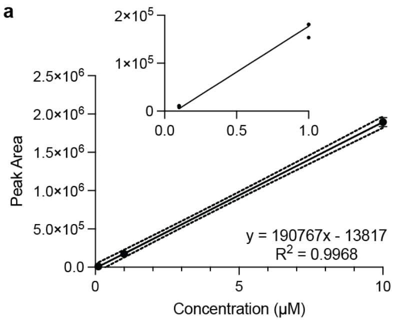

b

Area	Calculated concentration (μM)
230619	1
33978	0.3
206566	1
average	1

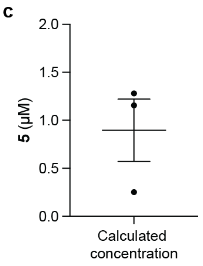

Supplementary Figure 10: Approximation of **5** in *N. tenerifensis* spent medium. **a)** Standard curve of **5** at 100 nM, 1 μM, 10 μM in molasses medium. Peak areas from DBCO-derivatized *N. tenerifensis* spent medium are highlighted in blue. Standards were run in biological triplicates. The dashed line indicates the 95% CI. Error bars indicate the SEM. **b)** Concentrations calculated from raw peak areas of *N. tenerifensis* spent medium. *N. tenerifensis* samples were run in biological triplicates. **c)** Concentration of **5** in *N. tenerifensis* supernatants. The mean and SEM are shown.

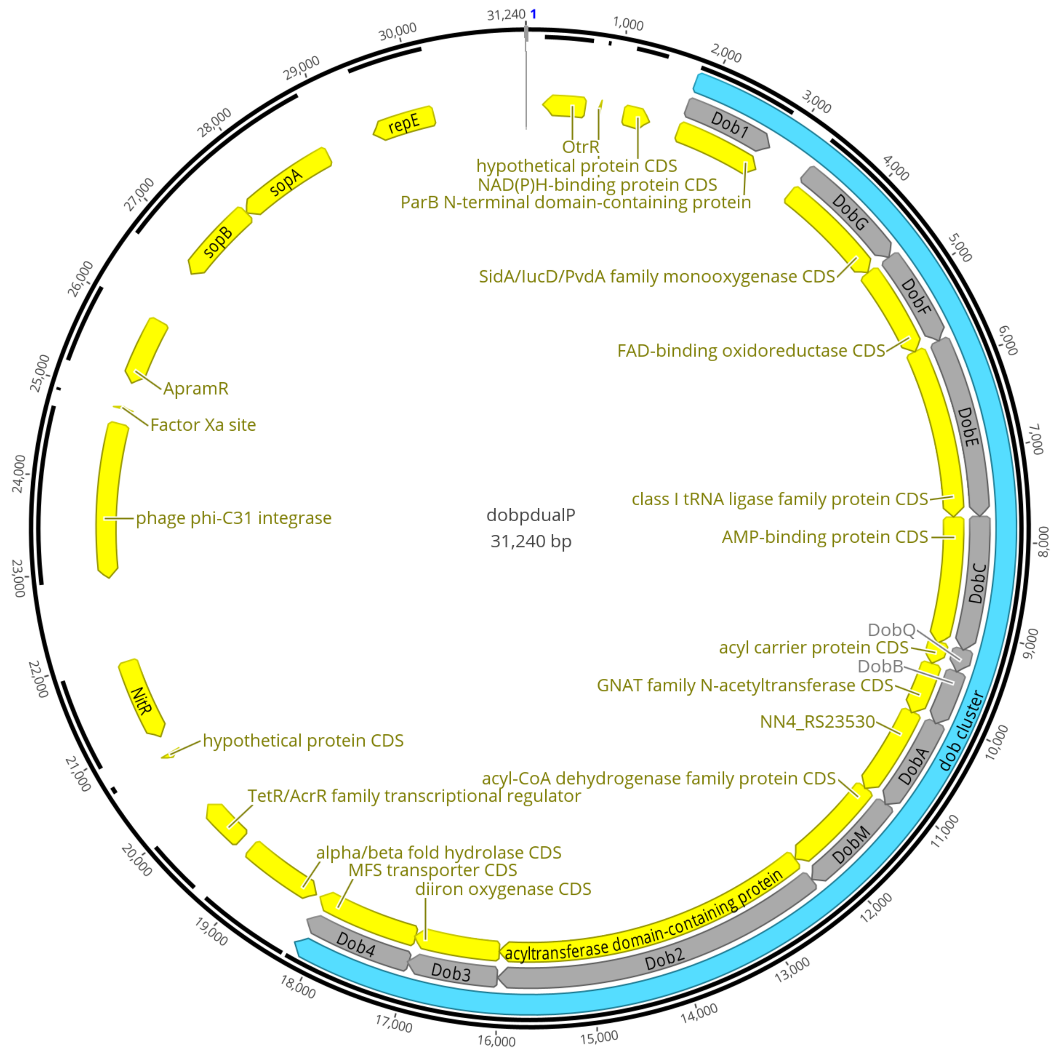

Supplementary Figure 11: Vector map of *dob*-pDualP.

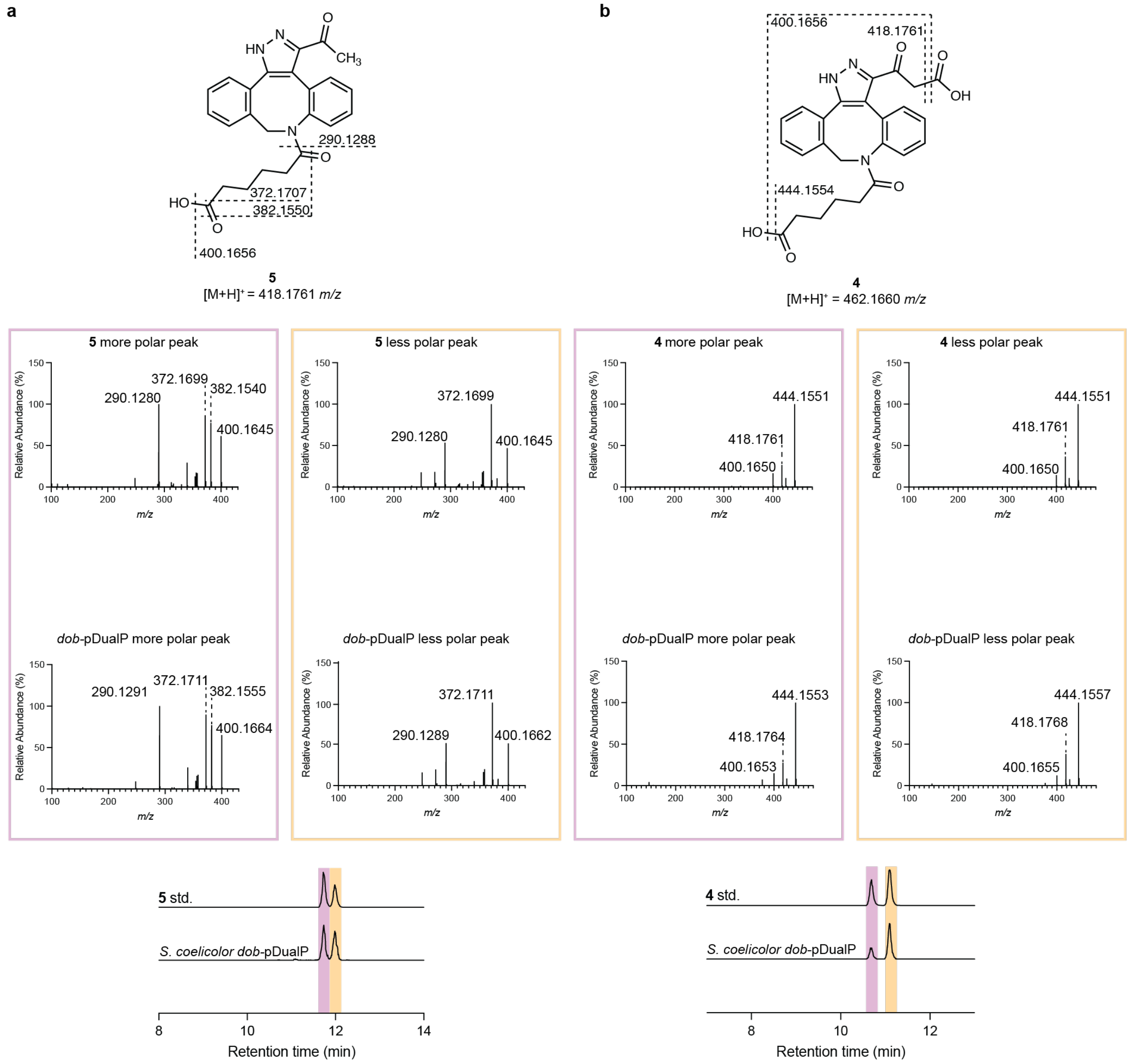

Supplementary Figure 12: MS/MS spectra of both regioisomers of synthetic **5** and **4**, and comparison with derivatized spent media from *S. coelicolor dob*-pDualP. **a)** Fragmentation pattern of DBCO-acid derivatized *S. coelicolor dob*-pDualP spent media matches that of synthetic **5.** EIC (*m/z* = 418.1761 ± 5 ppm) of the DBCO-acid derivatized *S. coelicolor dob*-pDualP spent media compared to a synthetic standard. **b)** Fragmentation pattern of DBCO-acid derivatized *S. coelicolor dob*-pDualP spent media matches that of synthetic **4**. **4** was prepared through incubation of **4-OMe** with PLE. EIC (*m/z* = 462.1660 ± 5 ppm) of the DBCO-acid derivatized *S. coelicolor dob*-pDualP spent media compared to a synthetic standard.

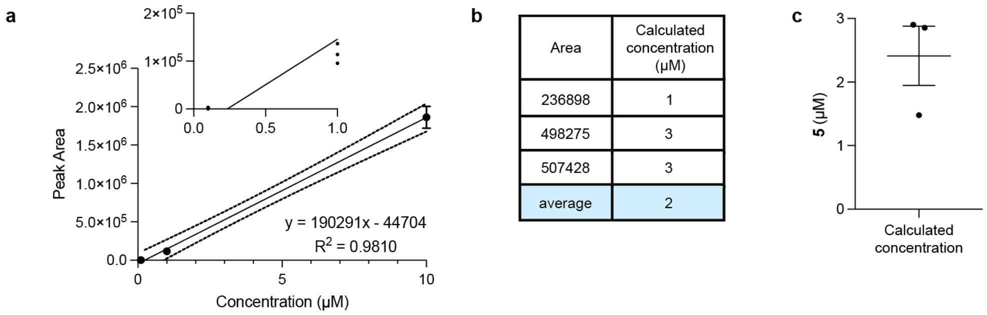

Area	Calculated concentration (µM)
236898	1
498275	3
507428	3
average	2

Supplementary Figure 13: Approximation of **5** in *S. coelicolor dob*-pDualP spent medium. **a)** Standard curve of **5** at 100 nM, 1 µM, 10 µM in ISP4 medium. Peak areas from DBCO-derivatized *S. coelicolor dob*-pDualP spent medium are highlighted in blue. Standards were run in biological triplicates. The dashed line indicates the 95% CI. Error bars indicate the SEM. **b)** Concentrations calculated from raw peak areas of *S. coelicolor dob*-pDualP spent medium. *S. coelicolor dob*-pDualP samples were run in biological triplicates. **c)** Concentration of **5** in *S. coelicolor dob*-pDualP supernatants. The mean and SEM are shown.

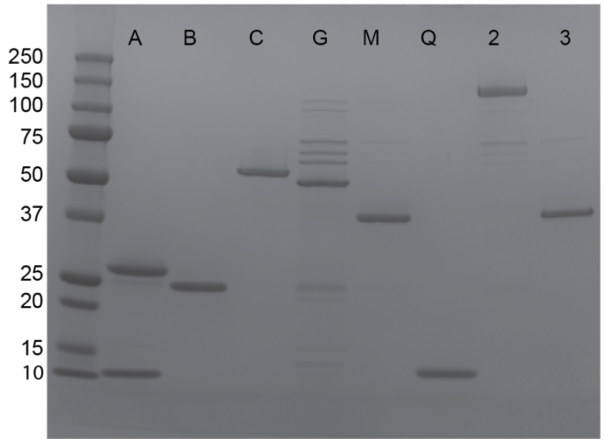

Supplementary Figure 14: SDS-PAGE gel of purified Dob biosynthetic enzymes. Molecular weights for His_6-tagged constructs were calculated using ExPASy ProtParam[45]: DobA (39.3 kDa; 12.1 kDa and 27.2 kDa after autoproteolysis at C112 to produce the mature C45 peptidase), DobB (25.1 kDa), DobC (56.1 kDa), DobG (51.8 kDa), DobM (43.7 kDa), DobQ (11.5 kDa), Dob2 (133.9 kDa), Dob3 (40.2 kDa). Precision Plus All Blue Molecular Weight Standard (Bio-Rad) was used as the ladder.

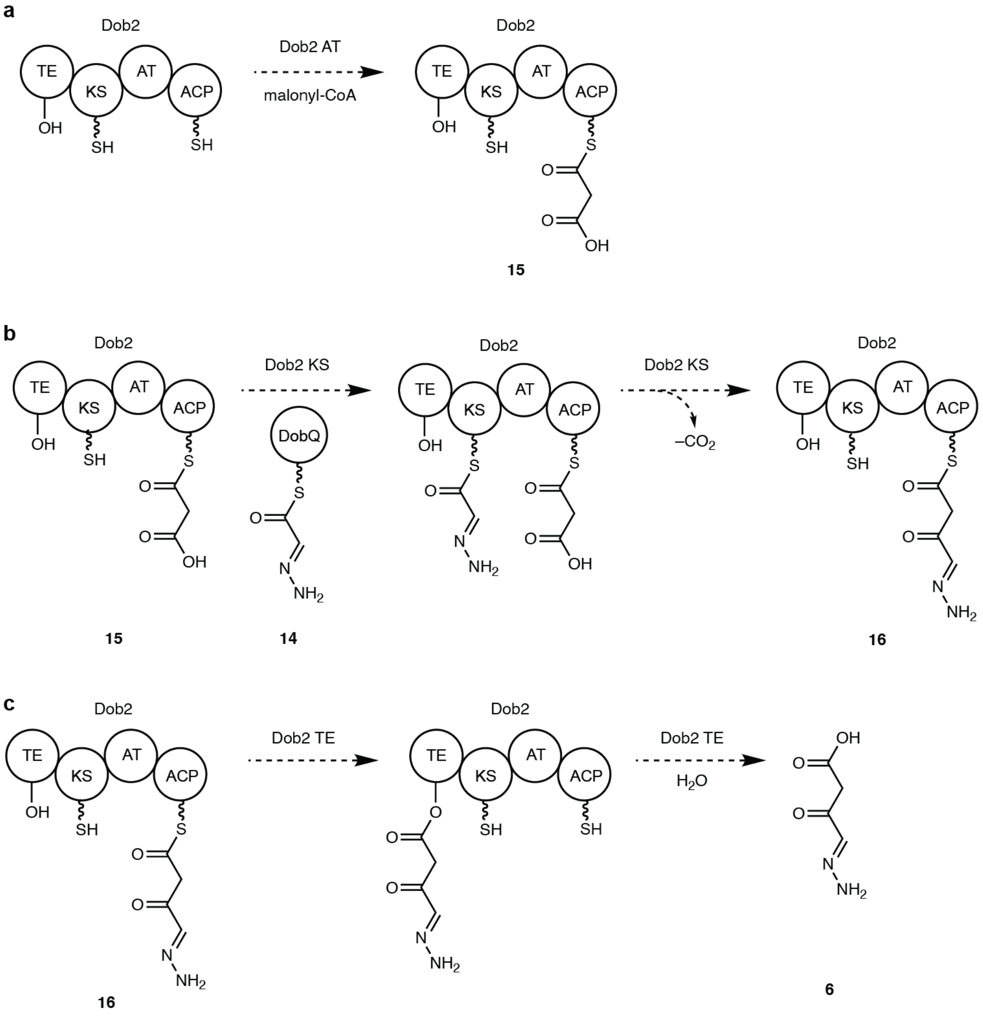

Supplementary Figure 15: Predicted functions of Dob2 domains. **a)** The Dob2 AT domain likely loads a malonyl extender unit. **b)** The KS domain likely catalyzes translocation of **14** from DobQ to a conserved Cys residue, followed by C–C bond-forming decarboxylative Claisen condensation between the malonyl extender unit and the starter unit **14** to produce **16**. **c)** The thioesterase domain likely translocates the mature PKS intermediate to a conserved serine residue (S88) prior to hydrolytic release of **6**.

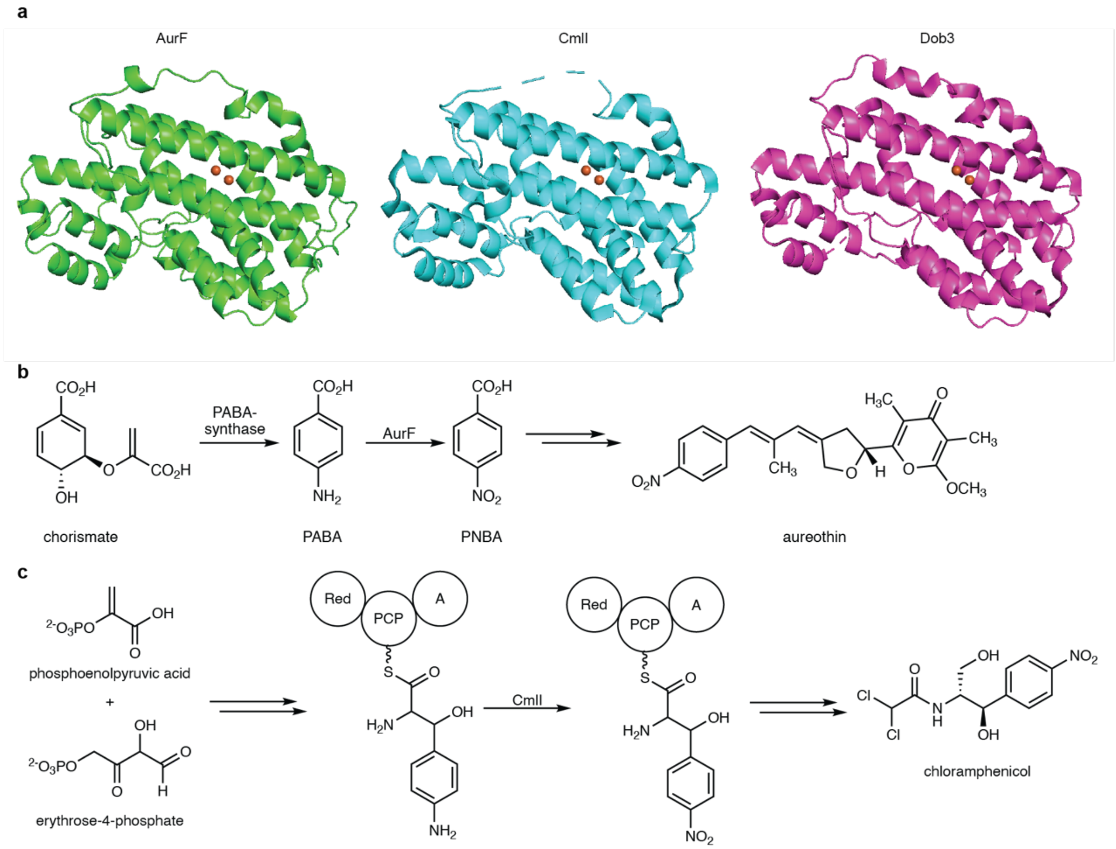

Supplementary Figure 16: Dob3 is structurally homologous to ferritin-like diiron *N*-oxygenases (FDOs) AurF and CmlI. **a)** Comparison of the AlphaFold predicted Dob3 structure to the X-ray crystal structures of AurF (PDB: 3CHH) and CmlI (PDB: 5HYH). Nitro-forming *N*-oxygenation reactions catalyzed by **b)** AurF[45] and **c)** CmlI[83] during the biosynthesis of aureothin and chloramphenicol, respectively.

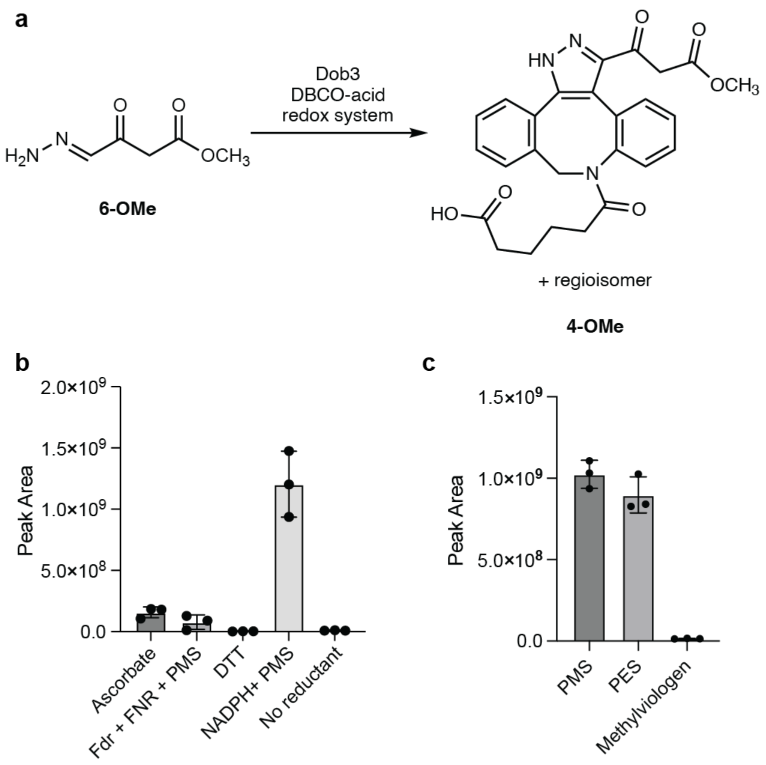

Supplementary Figure 17: Optimization of Dob3 redox system. **a)** Dob3 reaction scheme. **b)** Screening of redox systems demonstrates NADPH with PMS provides the greatest conversions. Fdr = Spinach ferredoxin, FNR = Spinach ferredoxin reductase, DTT = dithiothreitol. **c)** Screening of electron mediators in conjunction with NADPH demonstrates PMS provides the greatest conversions. PMS = phenazine methosulfate, PES = phenazine ethosulfate. All experiments were performed in biological triplicates. Error bars indicate mean ± standard deviation.

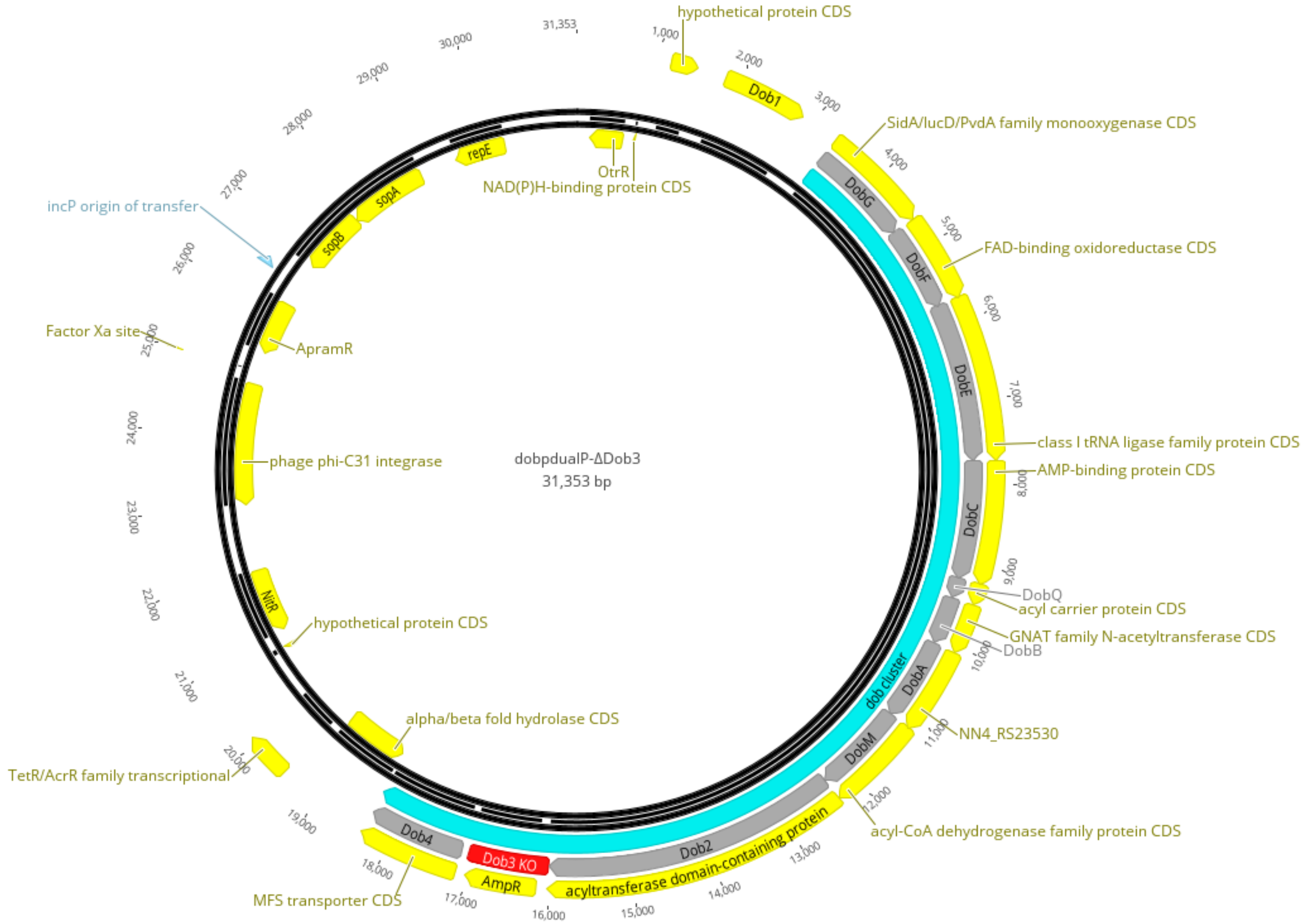

Supplementary Figure 18: Vector map of *dob*-pDualP Δ*dob3*.

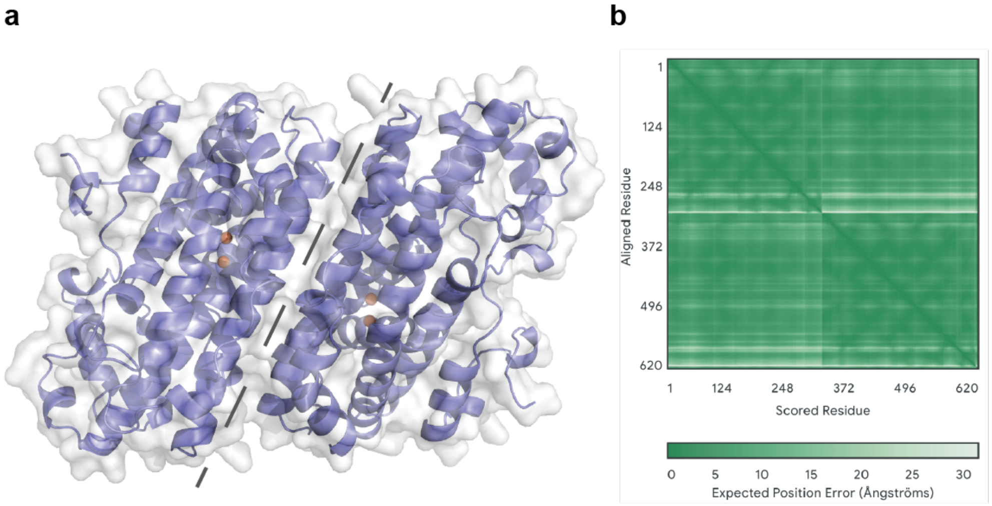

Supplementary Figure 19: AlphaFold 3 predicts that Dob3 is a homodimer. **a)** AlphaFold 3 predicted structure of the Dob3 homodimer. The dimer interface is marked with a dashed line. Residues 1-26 form a long, unfolded chain and are omitted for clarity. **b)** Expected Position Error Plot for the predicted Dob3 homodimer structure.

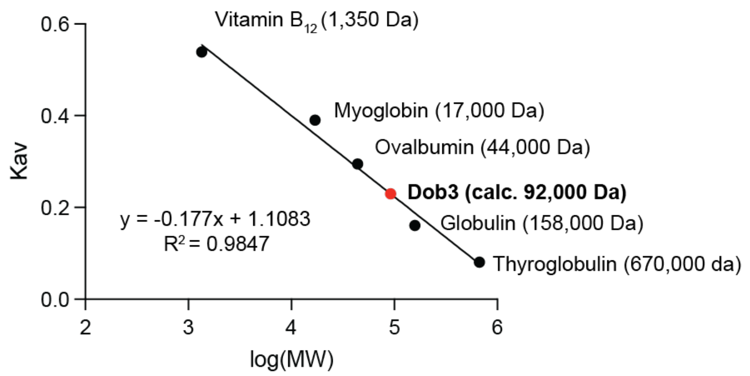

Supplementary Figure 20: Size exclusion chromatography is consistent with a Dob3 homodimer (MW_{dimer} = 80.4 kDa, MW_{calc} = 92.0 kDa).

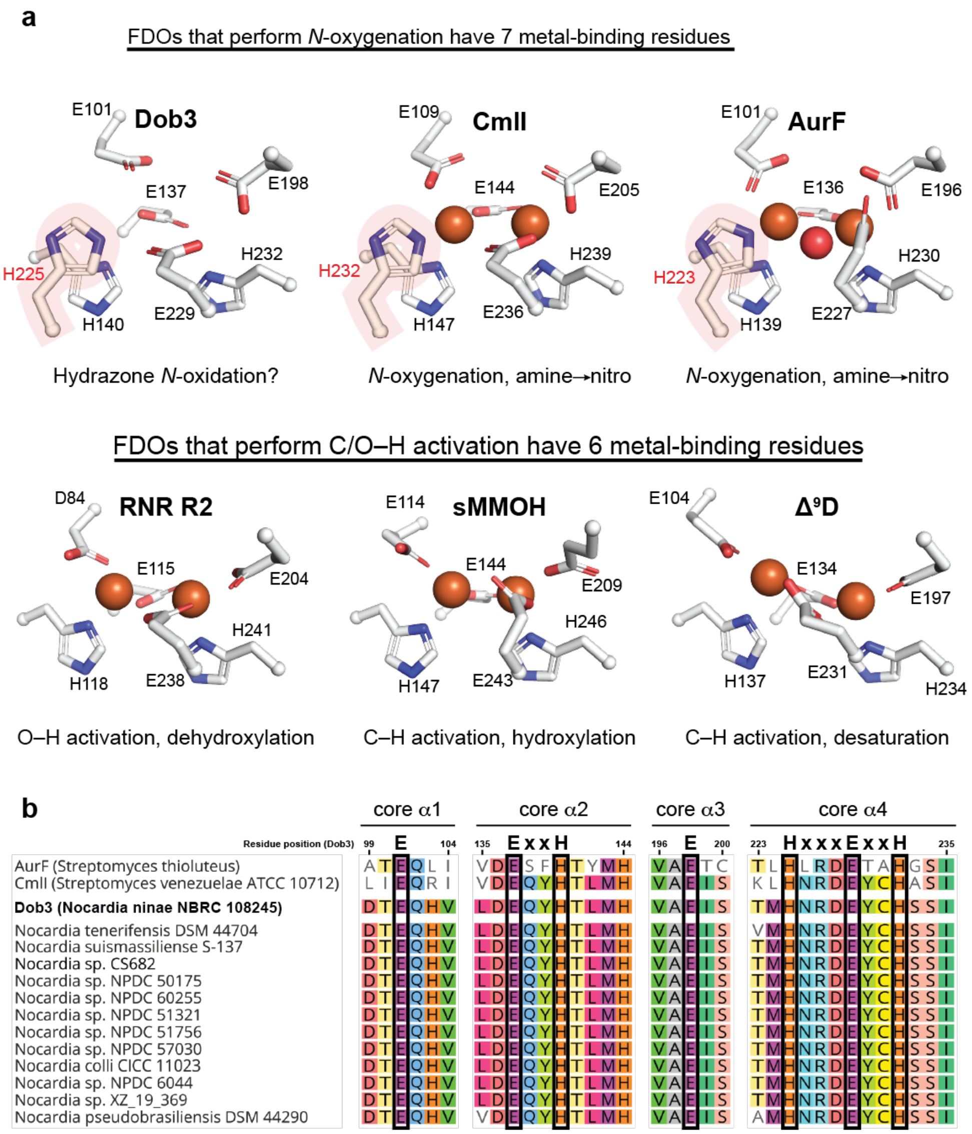

Supplementary Figure 21: Comparison of Dob3 to known FDOs suggests its activity as an *N*-oxygenase. **a)** The AlphaFold 3 predicted active site structure of Dob3 compared to the X-ray crystal structures of CmlI (PDB: 5HYH), AurF (PDB: 3CHH), RNR R2 (PDB: 1PIY), sMMOH (PDB: 6YD0), and Δ^9-stearoyl-ACP desaturase (PDB: 1AFR). **b)** Multiple sequence alignment of Dob3 and its homologs from *Nocardia* strains with the FDO *N*-oxygenases CmlI and AurF demonstrates conservation of Fe-binding (boxed) residues.

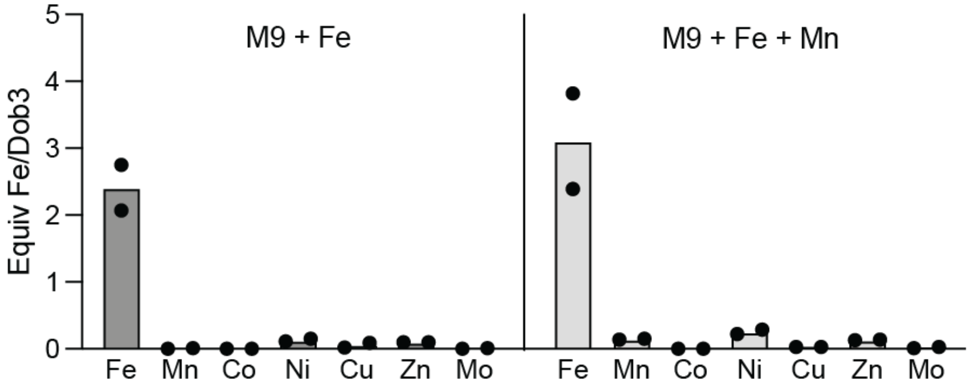

Supplementary Figure 22: ICP-MS reveals Fe is the most abundant metal associated with Dob3 when expressed in M9 medium supplemented with either Fe or Fe + Mn. Experiments were performed in biological duplicates.

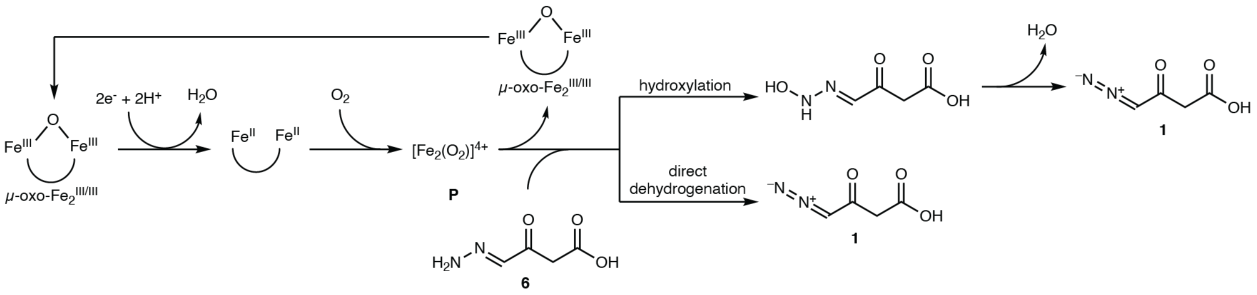

Supplementary Figure 23: Proposed mechanism of Dob3. Analogous to the mechanism of AurF, the μ-oxo-diferric cofactor is reduced to the diferrous state and subsequent oxidation by molecular oxygen yields a reactive differic-peroxo intermediate (**P**).[84,85] Hydroxylation and subsequent dehydration, or direct dehydrogenation of **6** yields **1**.

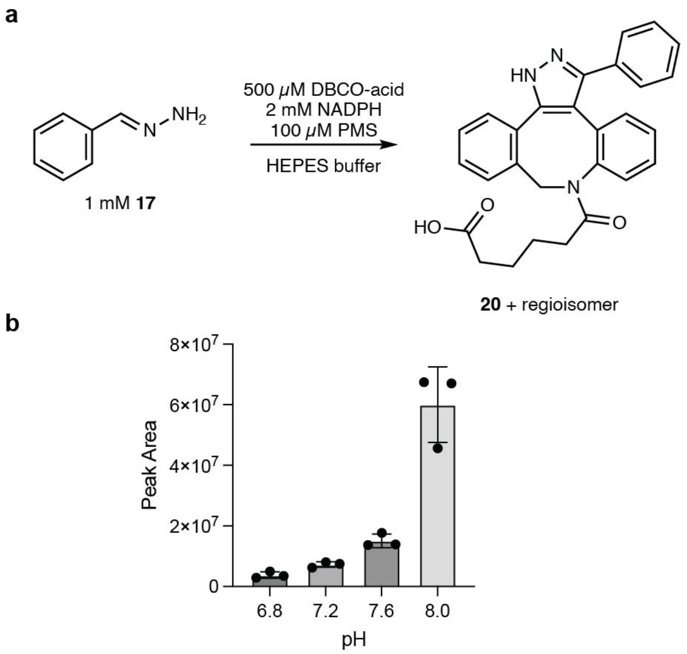

Supplementary Figure 24: pH dependence of autoxidation of **17**. **a)** Reaction scheme. **b)** Combined LC–MS peak areas of both regioisomers of **20.** Experiments were performed in biological triplicates. Error bars indicate mean ± standard deviation. Product identity was confirmed by comparison of LC–MS/MS data to a synthetic standard of **20**.

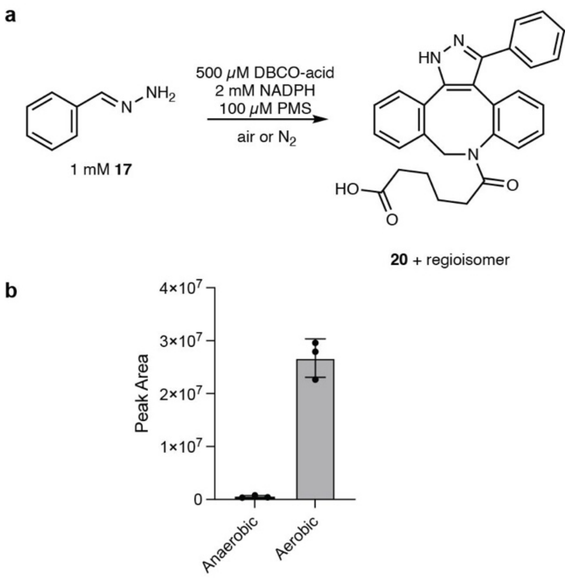

Supplementary Figure 25: Aerobic incubation of **17** with DBCO-acid, PMS, and NADPH yields more product than anaerobic incubations. **a)** Reaction scheme. Reaction mixtures were incubated at pH 8.0. **b)** Combined LC–MS peak areas of both regioisomers of **20**. Experiments were performed in biological triplicates. Error bars indicate mean ± standard deviation. Product identity was confirmed by comparison to a synthetic standard of **20**.

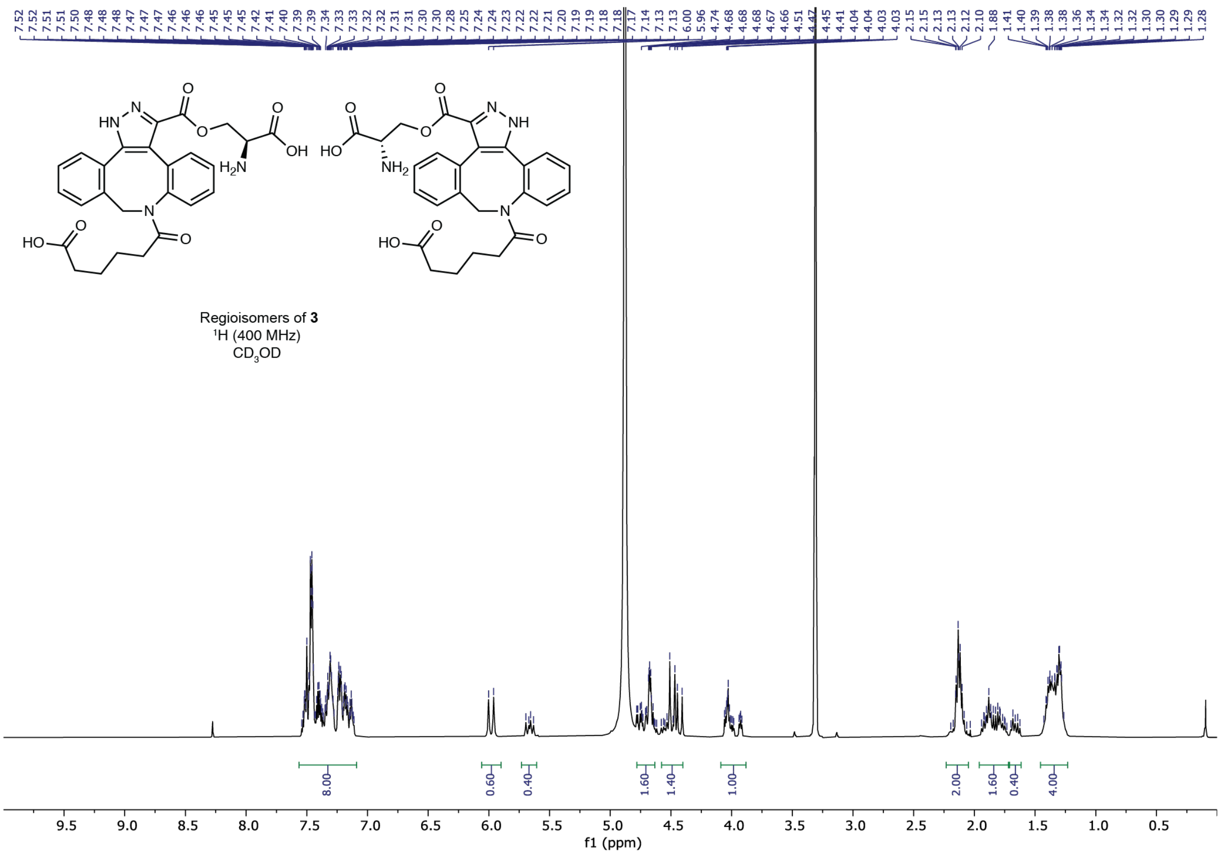

Supplementary Figure **26**: ^{1}H NMR spectrum of regioisomers of **3**.

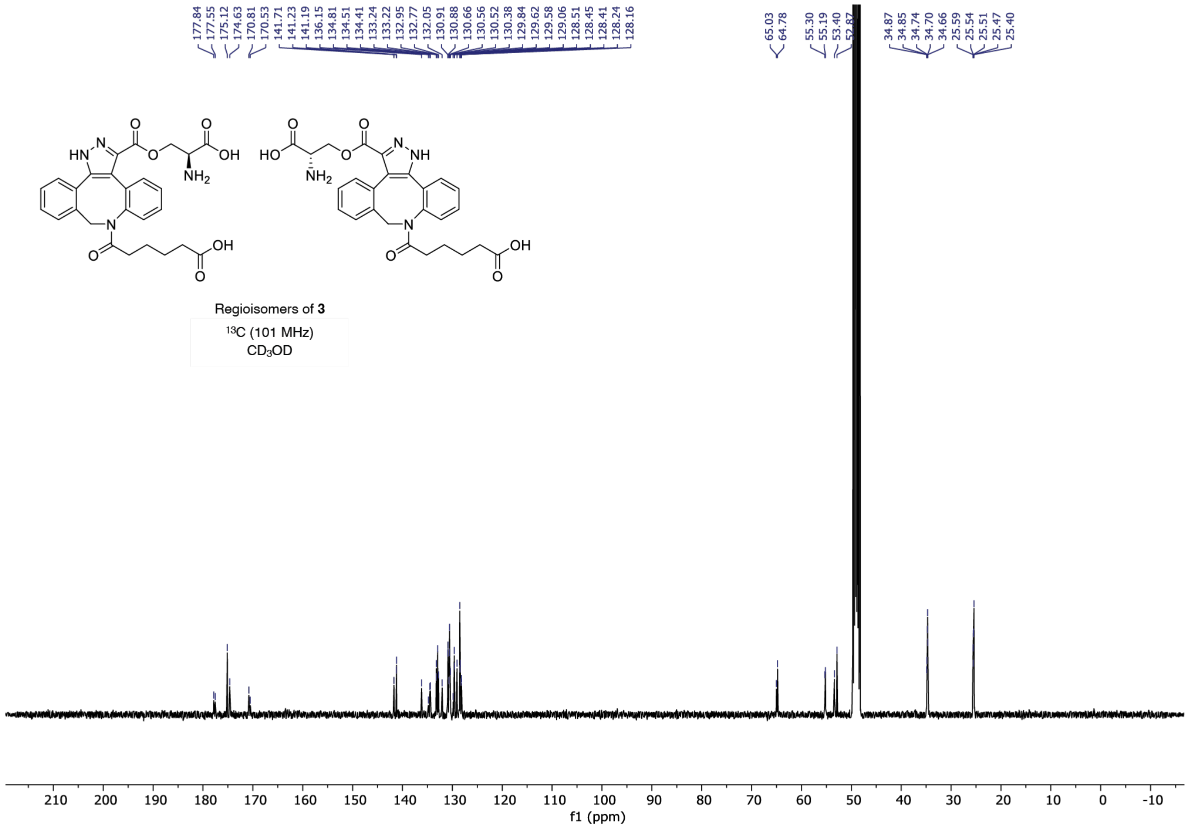

Supplementary Figure 27: ^{13}C NMR spectrum of **3** regioisomers.

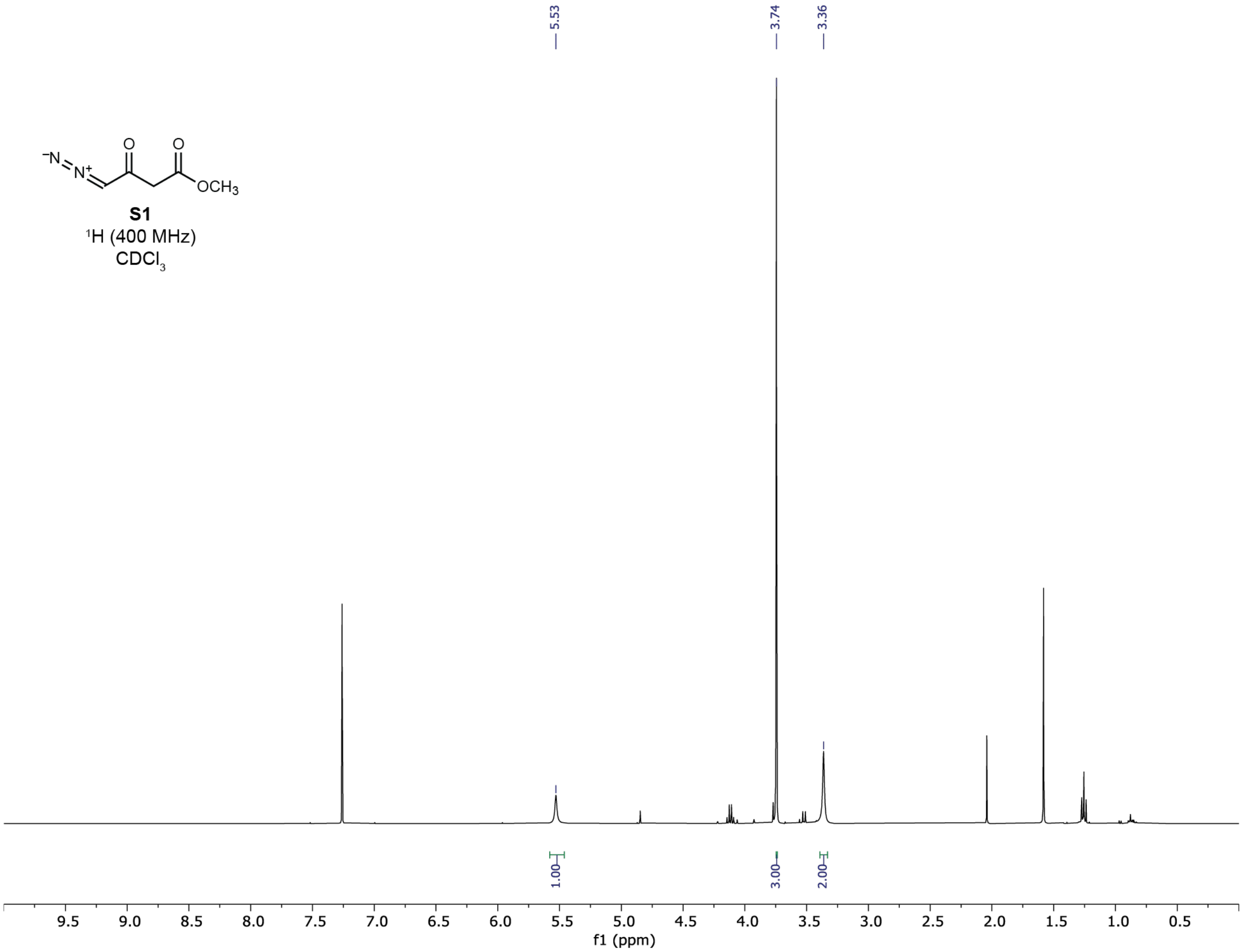

Supplementary Figure 28: ^{1}H NMR spectrum of **S1.**

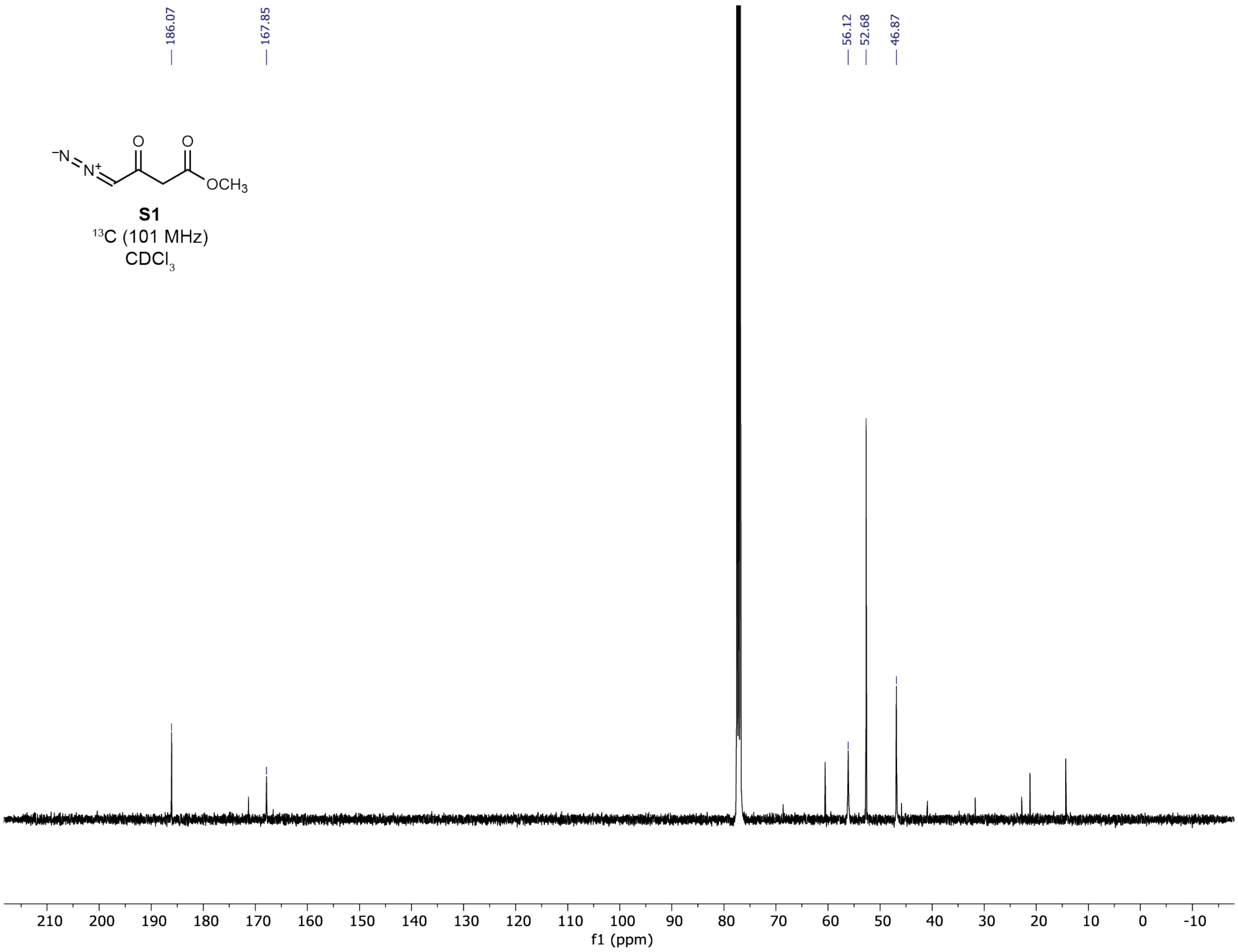

Supplementary Figure 29: ^{13}C NMR spectrum of **S1.**

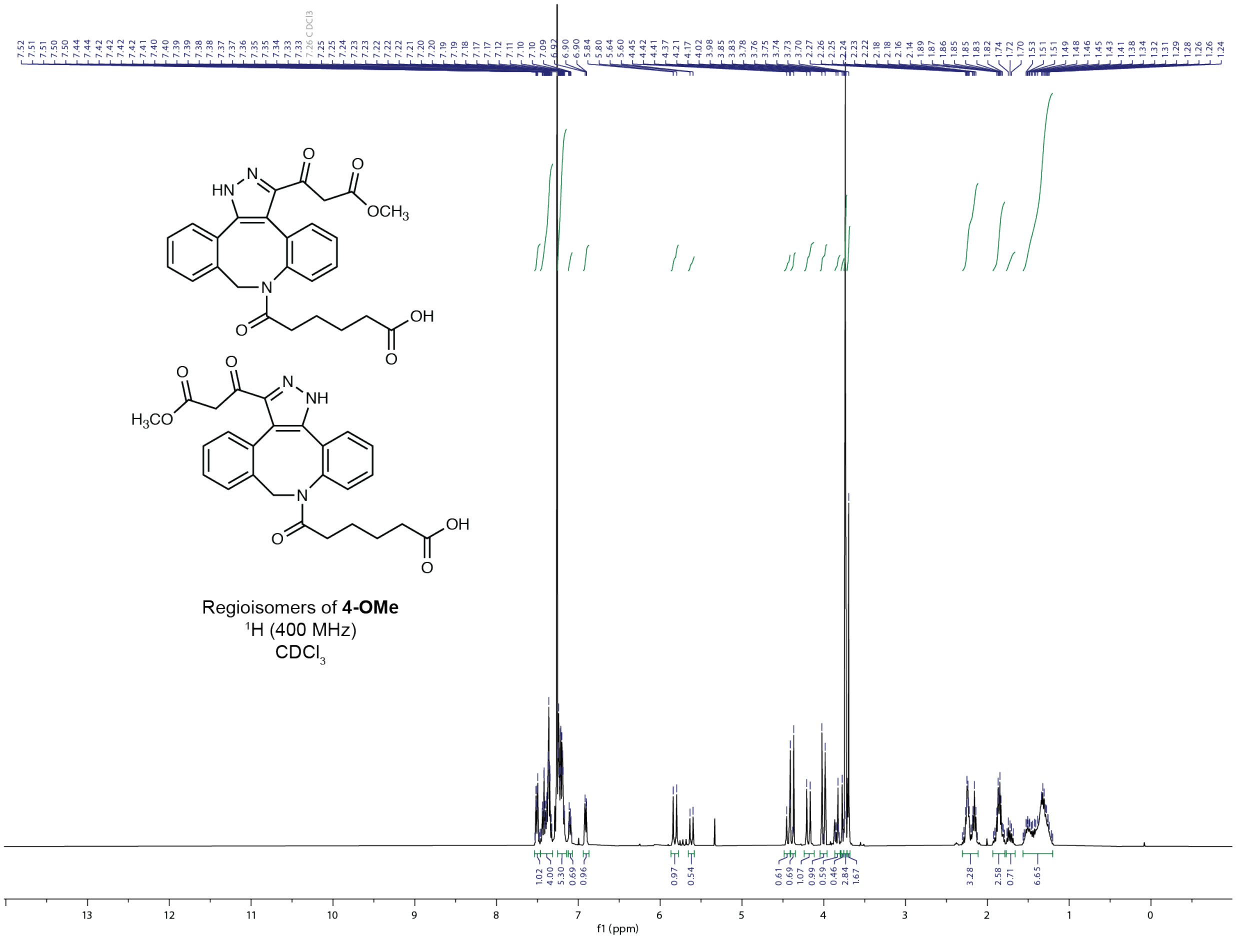

Supplementary Figure 30: ^{1}H NMR spectrum of regioisomers of **4-OMe**.

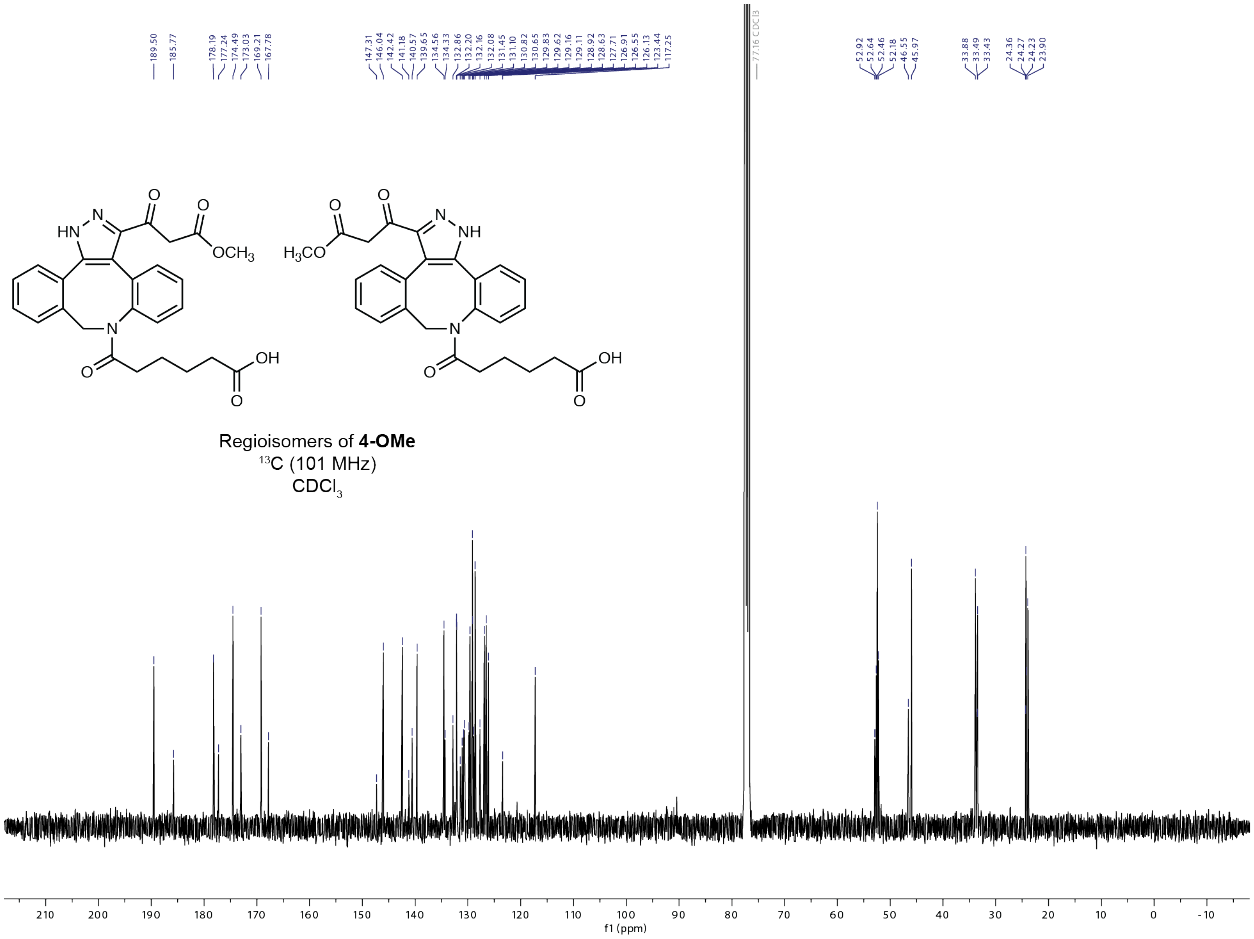

Supplementary Figure 31: ^{13}C NMR spectrum of regioisomers of **4-OMe**.

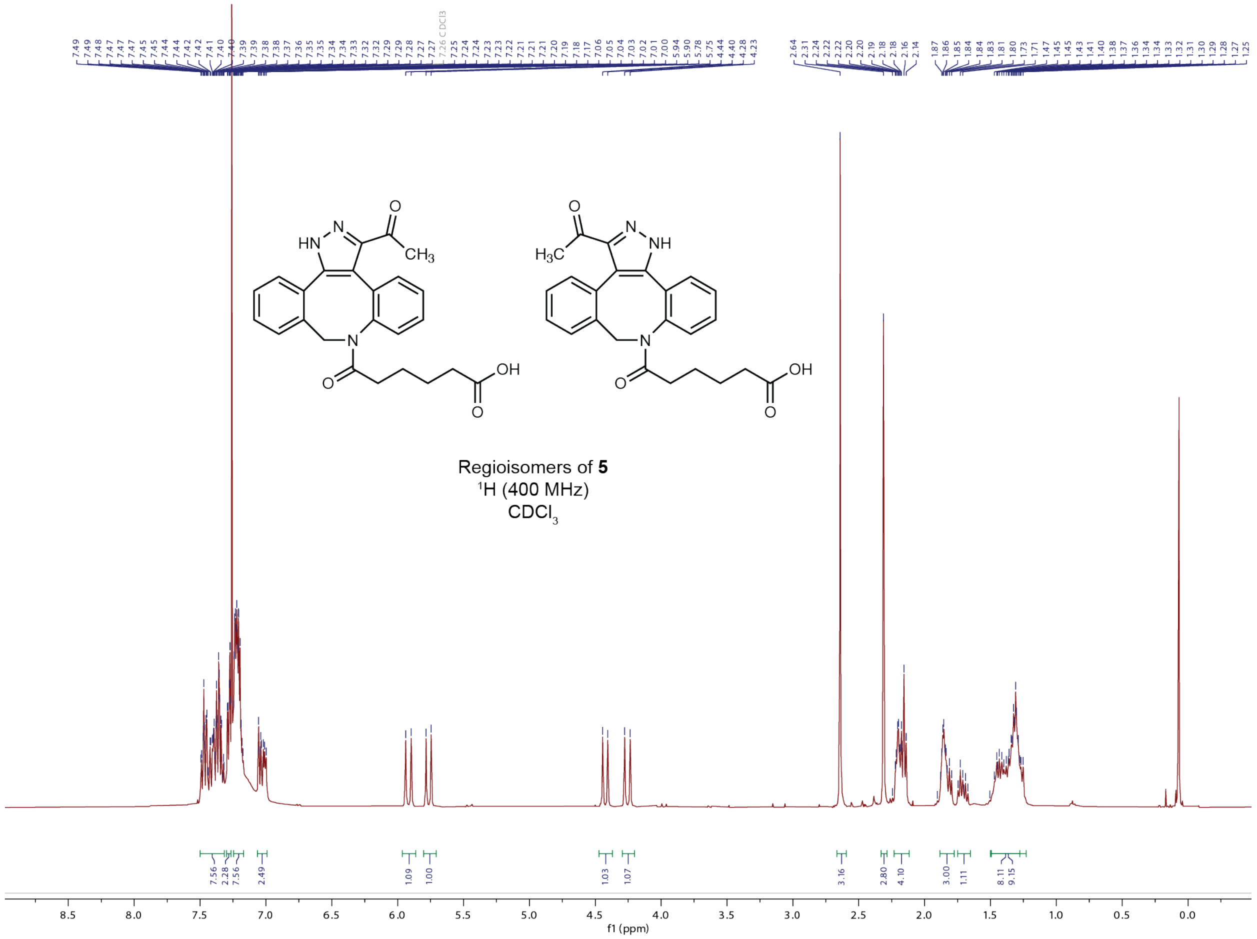

Supplementary Figure 32: ^{1}H NMR spectrum of regioisomers of **5**.

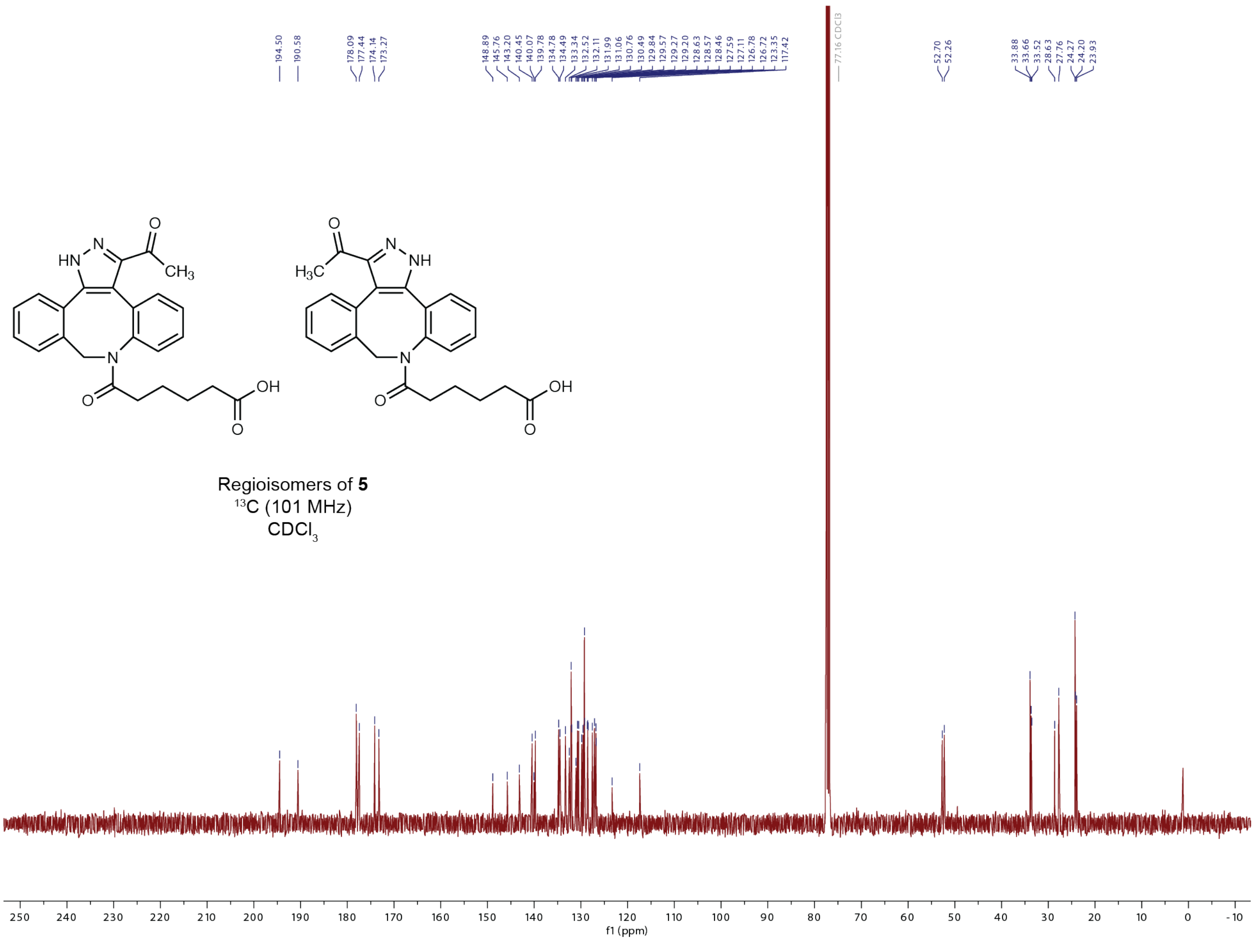

Supplementary Figure 33: ^{13}C NMR spectrum of regioisomers of **5**.

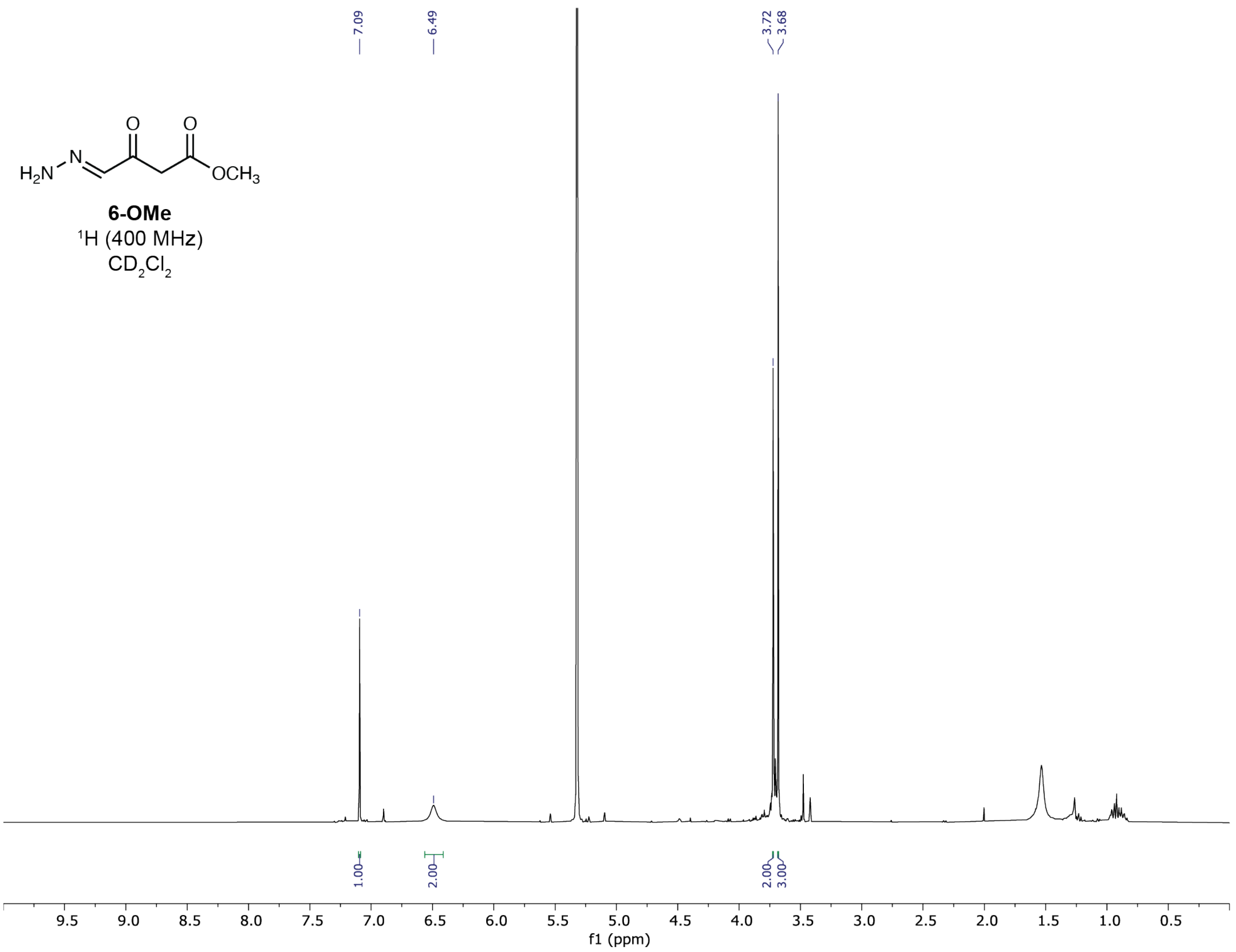

Supplementary Figure 34: ^{1}H NMR spectrum of **6-OMe.**

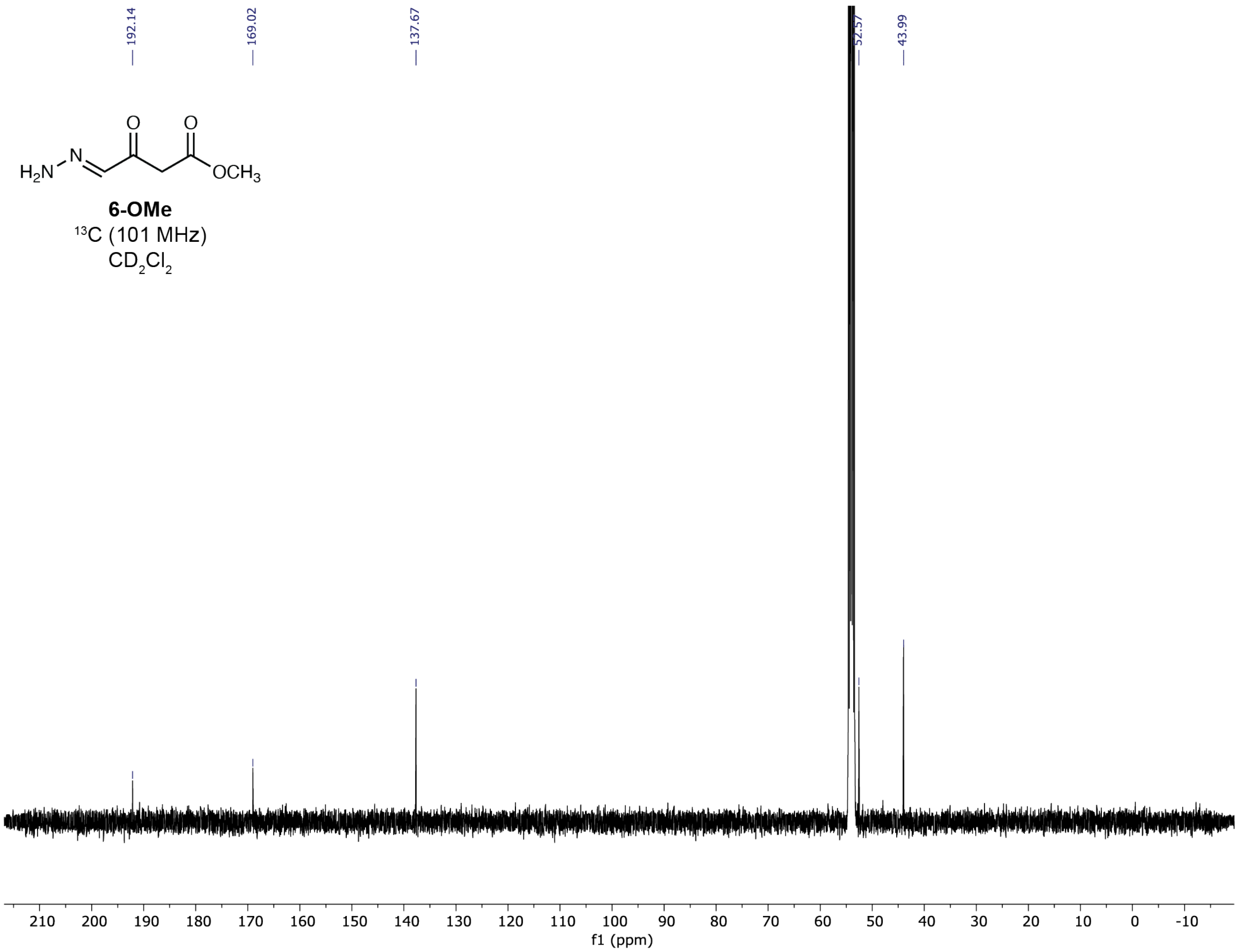

Supplementary Figure 35: ^{13}C NMR spectrum of **6-OMe.**

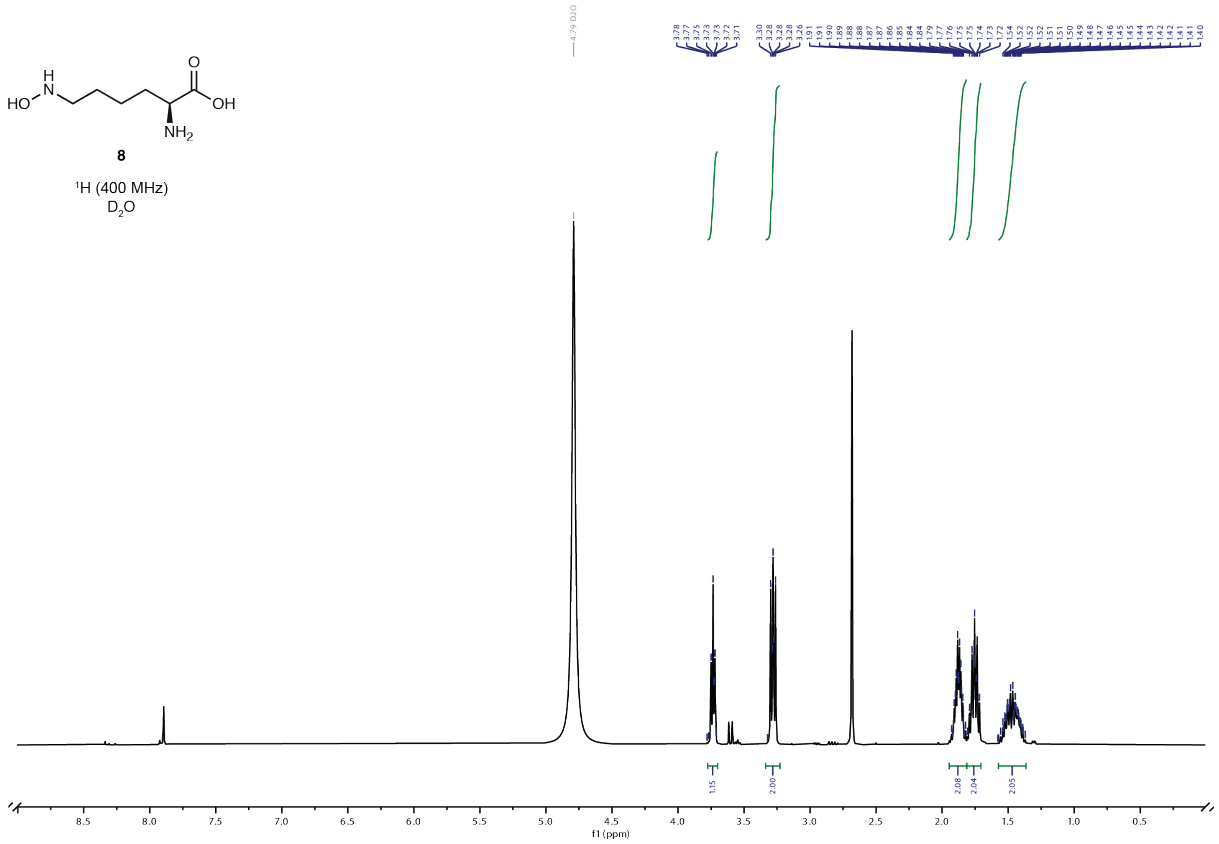

Supplementary Figure 36: ^{1}H NMR spectrum of **8.**

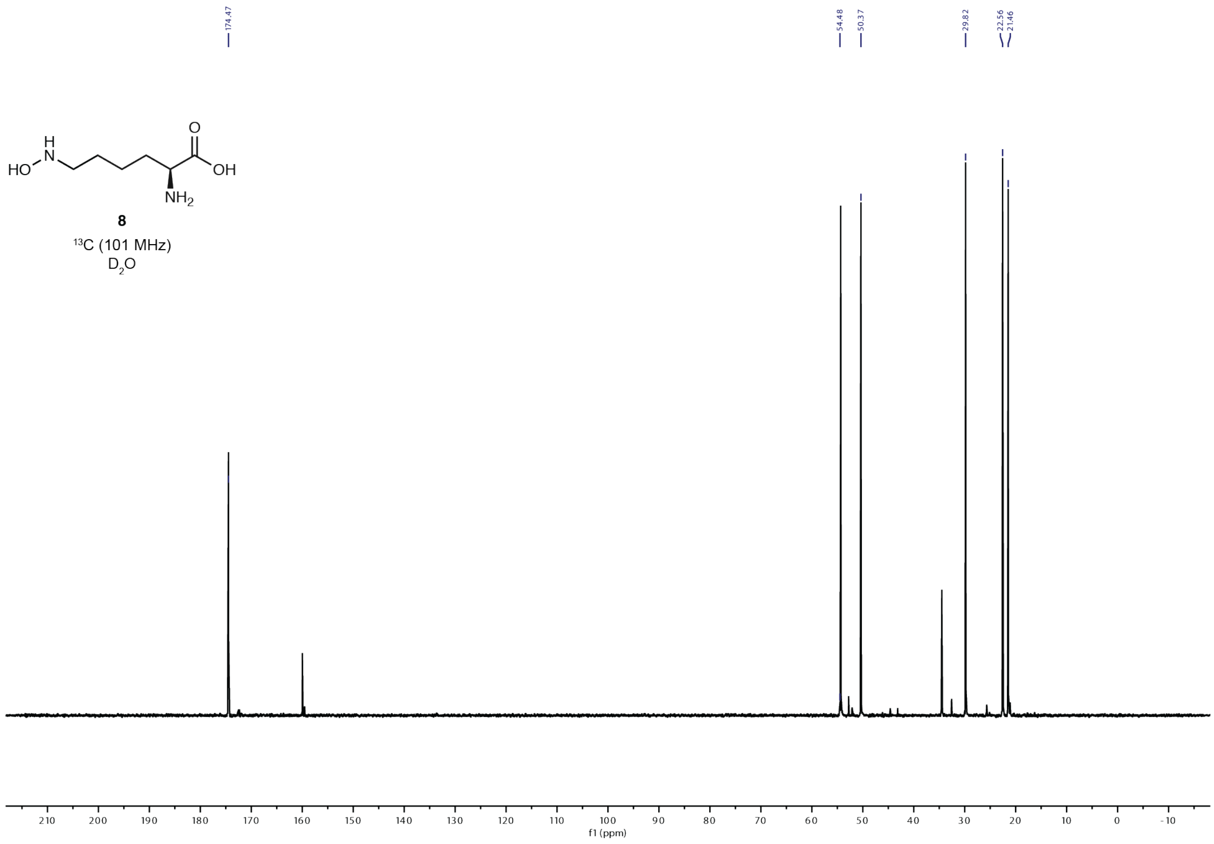

Supplementary Figure 37: ^{13}C NMR spectrum of **8.**

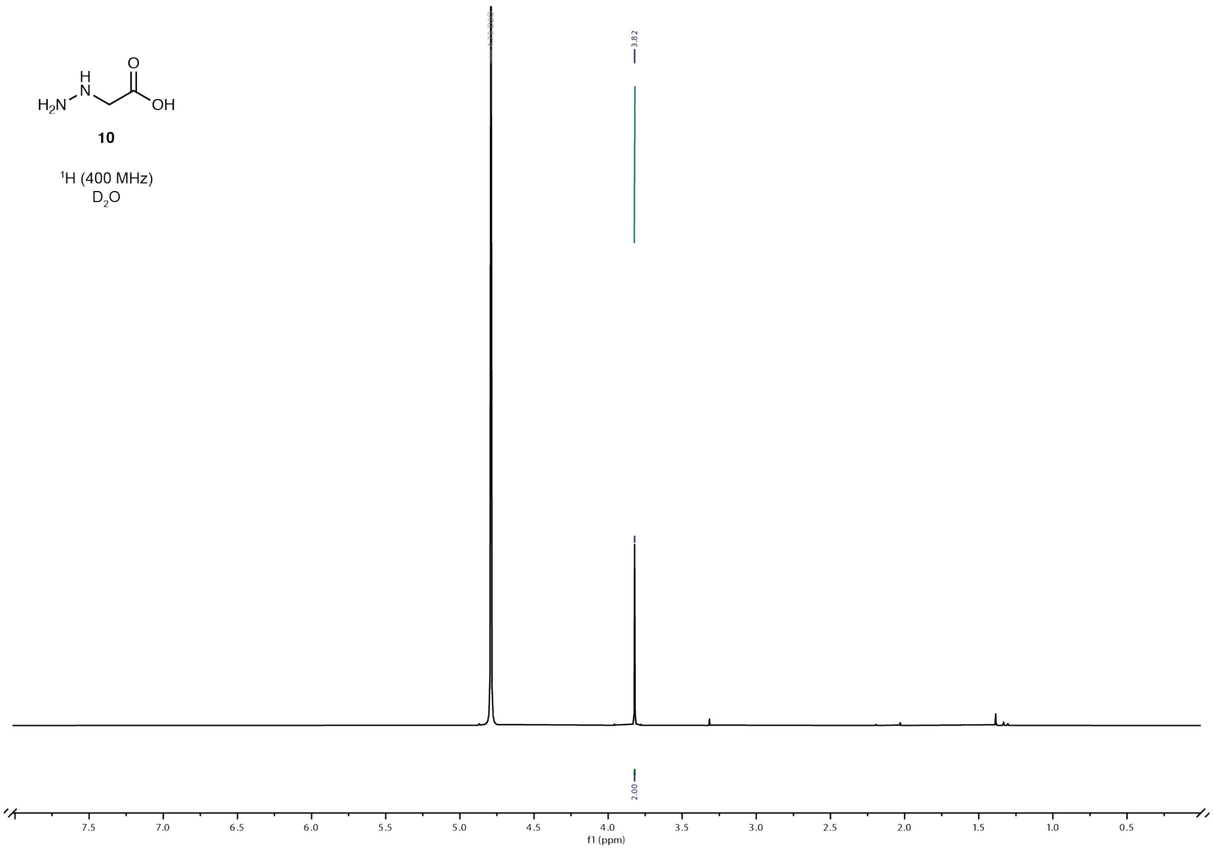

Supplementary Figure 38: ^{1}H NMR spectrum of **10.**

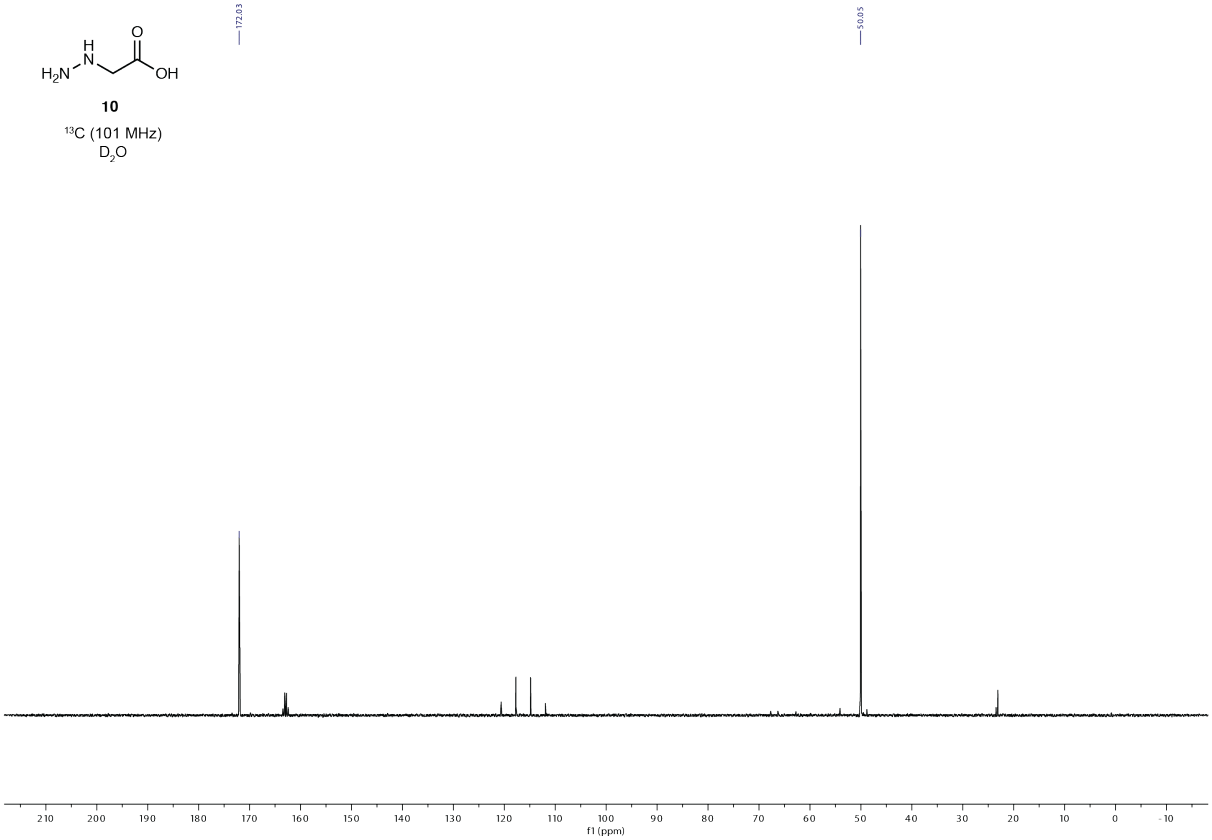

Supplementary Figure 39: ^{13}C NMR spectrum of **10.**

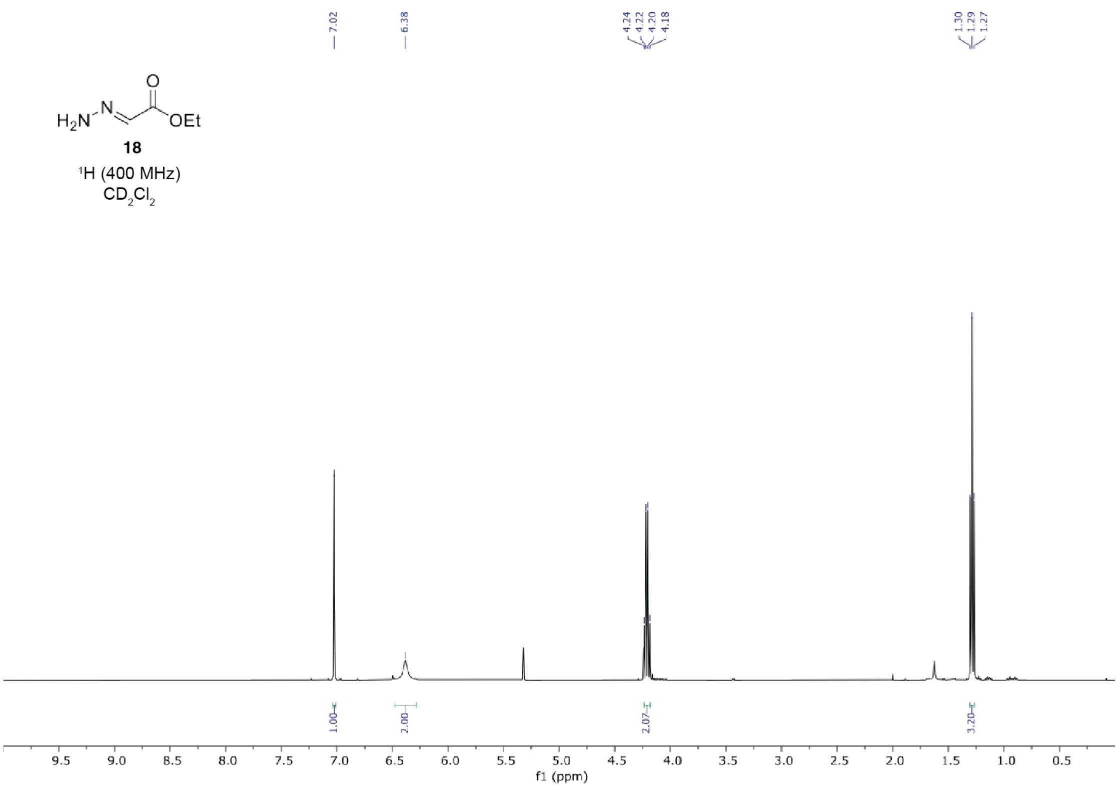

Supplementary Figure 40: ^{1}H NMR spectrum of **18.**

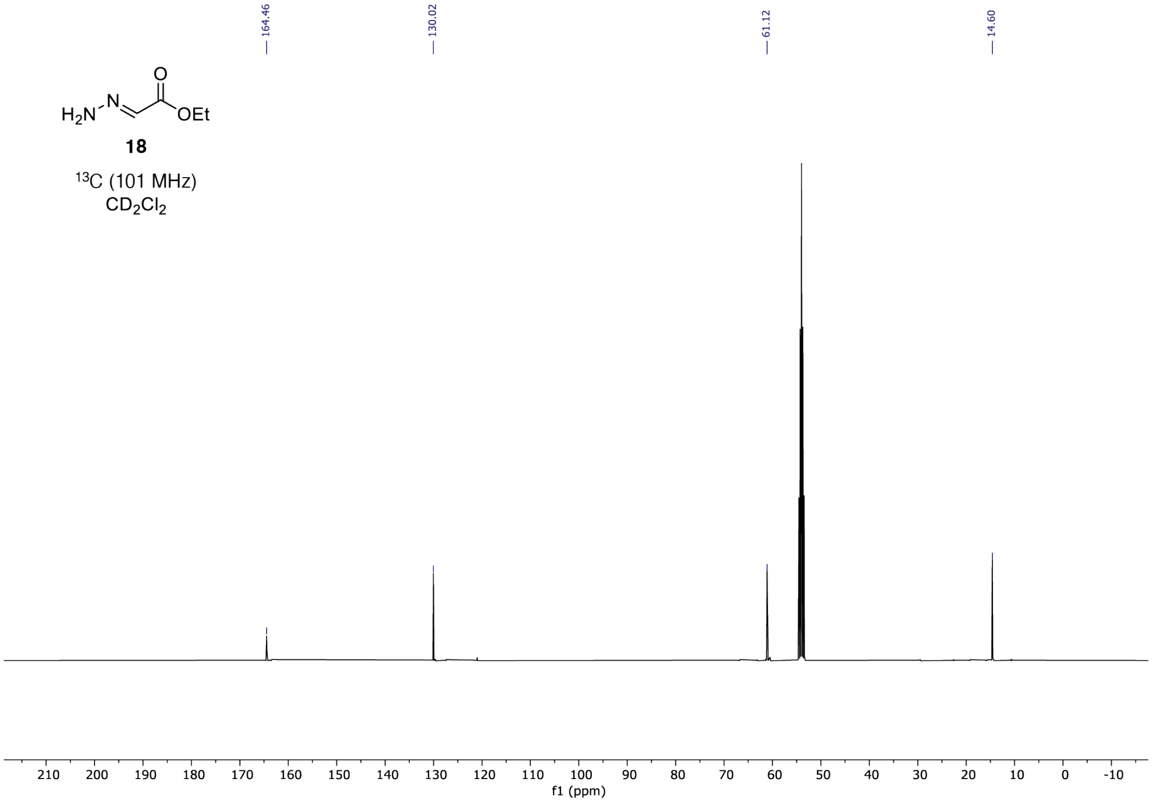

Supplementary Figure 41: ^{13}C NMR spectrum of **18.**

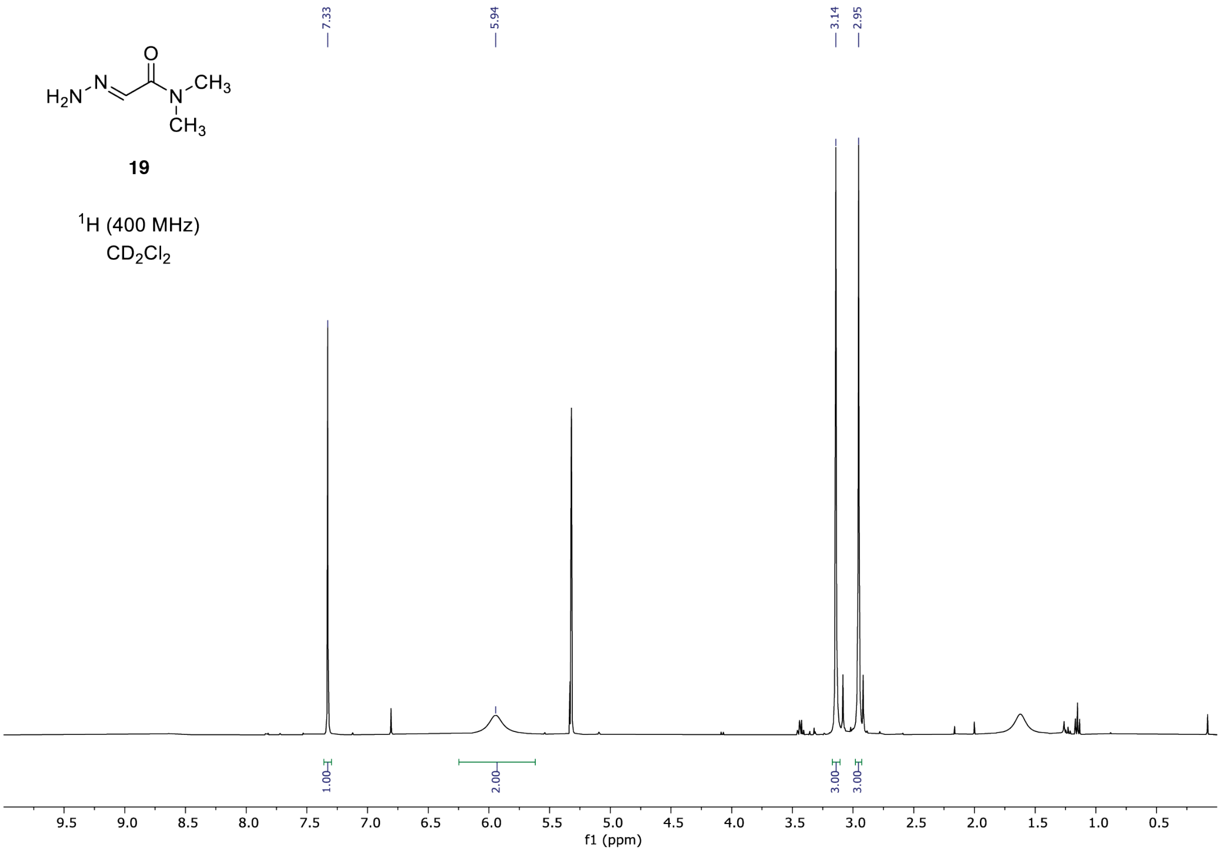

Supplementary Figure 42: ^{1}H NMR spectrum of **19.**

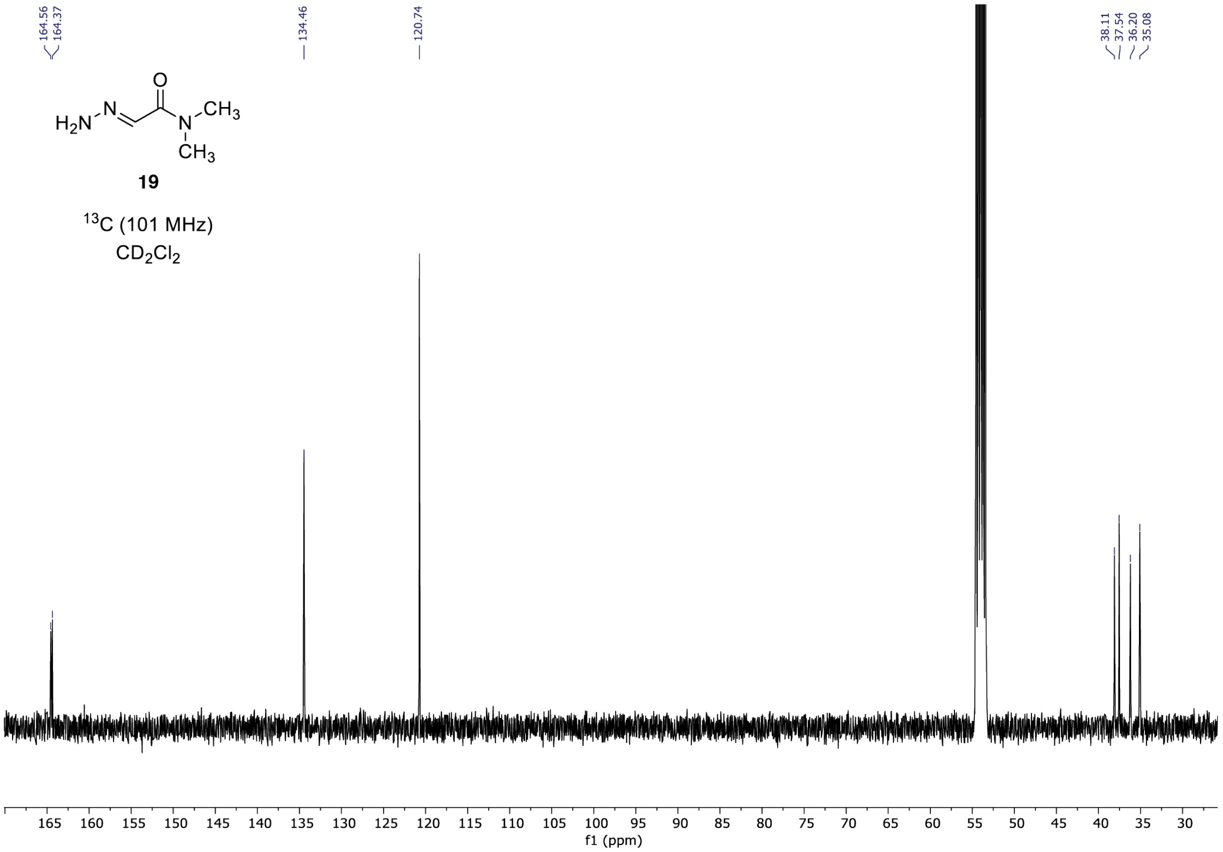

Supplementary Figure 43: ^{13}C NMR spectrum of **19**.

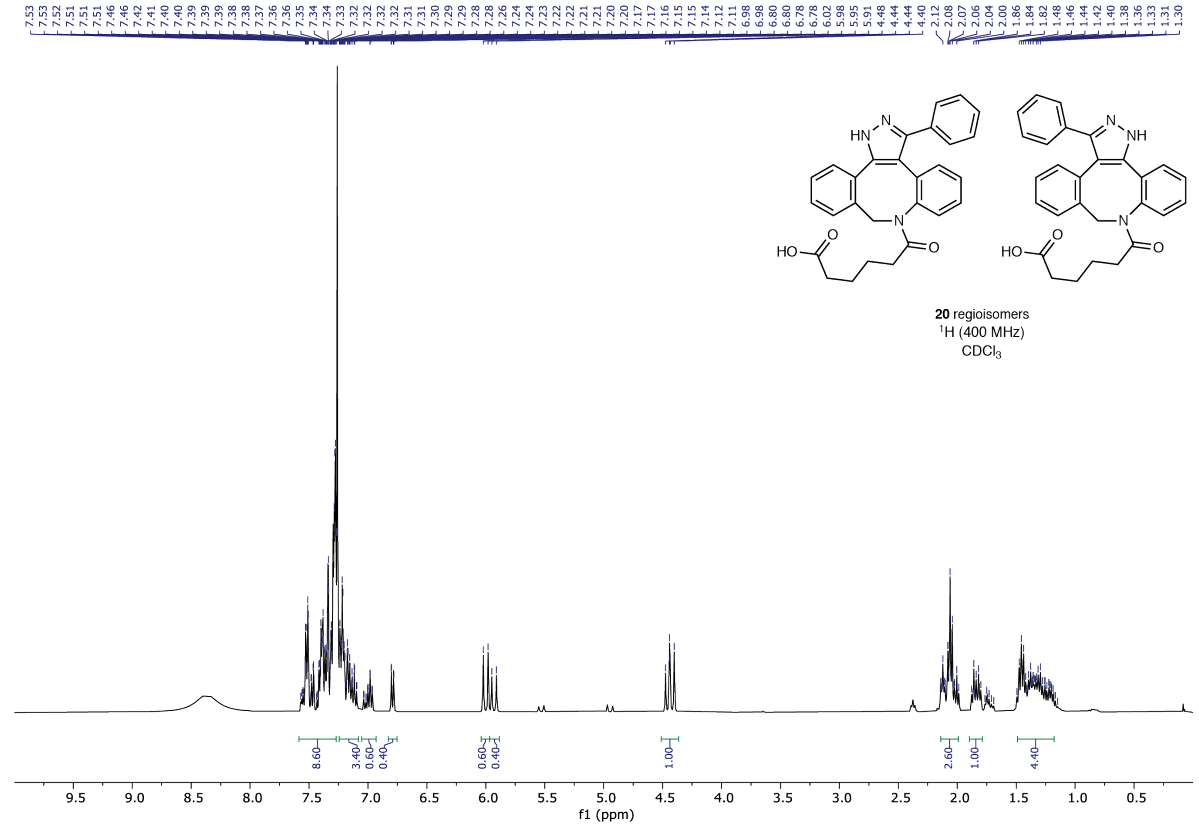

Supplementary Figure 44: ^{1}H NMR spectrum of **20**.

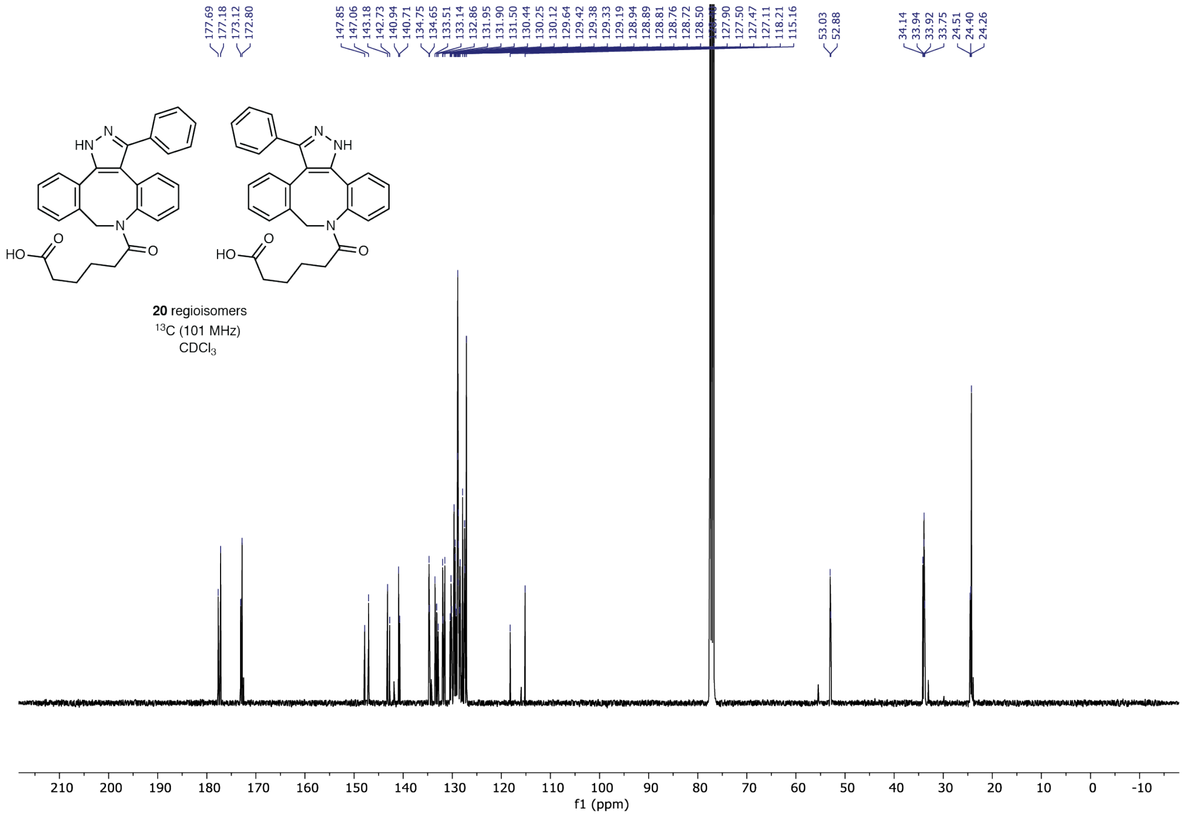

Supplementary Figure 45: ^{13}C NMR spectrum of **20**.

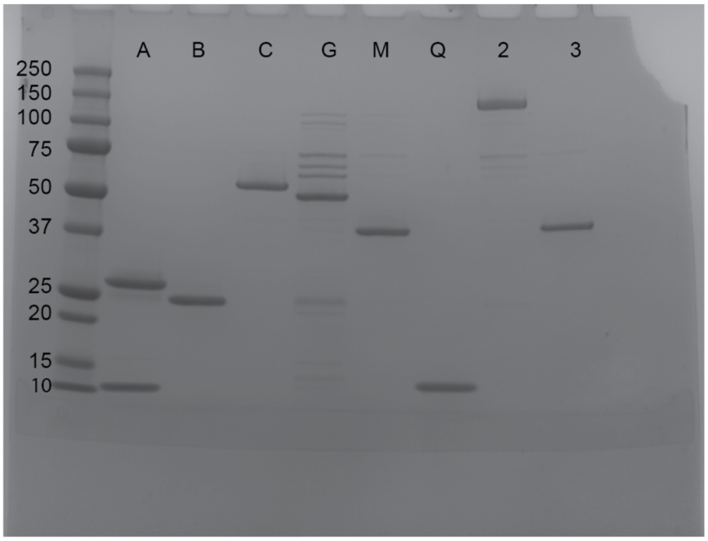

Supplementary Figure 46: Raw gel image.

References

71. Diazald® and Diazomethane Generators. *Sigma Aldrich Tech. Bull.* 1–6.

72. Pace, V., Verniest, G., Sinisterra, J.-V., Alcántara, A. R. & De Kimpe, N. Improved Arndt−Eistert Synthesis of α-Diazoketones Requiring Minimal Diazomethane in the Presence of Calcium Oxide as Acid Scavenger. *J. Org. Chem.* **75**, 5760–5763 (2010).

73. Yasui, E., Wada, M. & Takamura, N. New Entry for Synthesis of N-Acylhydrazones, Pyridazinones, and 1,3,4-Oxadiazin-6-ones from α-Amino Acid Esters. *Chem. Pharm. Bull. (Tokyo)* **55**, 1652–1654 (2007).

74. Döben, N., Yan, H., Kischkewitz, M., Mao, J. & Studer, A. Intermolecular Acetoxyaminoalkylation of α-Diazo Amides with (Diacetoxyiodo)benzene and Amines. *Org. Lett.* **20**, 7933–7936 (2018).

75. Javed, M. I. & Brewer, M. Diazo Preparation via Dehydrogenation of Hydrazones with “Activated” DMSO. *Org. Lett.* **9**, 1789–1792 (2007).

76. Choirunnisa, A. R. *et al.* New azodyrecins identified by a genome mining-directed reactivity-based screening. *Beilstein J. Org. Chem.* **18**, 1017–1025 (2022).

77. Molloy, E. M., Tietz, J. I., Blair, P. M. & Mitchell, D. A. Biological characterization of the hygrobafilomycin antibiotic JBIR-100 and bioinformatic insights into the hygrolide family of natural products. *Bioorg. Med. Chem.* **24**, 6276–6290 (2016).

78. Zhang, Y., Chen, C., Zhang, Y.-L., Kong, L.-Y. & Luo, J.-G. Target discovery of cytotoxic withanolides from Physalis angulata var. villosa via reactivity-based screening. *J. Pharm. Biomed. Anal.* **151**, 194–199 (2018).

79. Harris, L. A. *et al.* Reactivity-Based Screening for Citrulline-Containing Natural Products Reveals a Family of Bacterial Peptidyl Arginine Deiminases. *ACS Chem. Biol.* **15**, 3167–3175 (2020).

80. Castro-Falcón, G., Hahn, D., Reimer, D. & Hughes, C. C. Thiol Probes To Detect Electrophilic Natural Products Based on Their Mechanism of Action. *ACS Chem. Biol.* **11**, 2328–2336 (2016).

81. Li, J. *et al.* Identification and isolation of lantibiotics from culture: a bioorthogonal chemistry approach. *Org. Biomol. Chem.* **10**, 8677–8683 (2012).

82. Gasteiger, E. *et al.* Protein Identification and Analysis Tools on the ExPASy Server. in *The Proteomics Protocols Handbook* (ed. Walker, J. M.) 571–607 (Humana Press, Totowa, NJ, 2005). doi:10.1385/1-59259-890-0:571.

83. Lu, H., Chanco, E. & Zhao, H. CmlI is an N-oxygenase in the biosynthesis of chloramphenicol. *Tetrahedron* **68**, 10.1016/j.tet.2012.06.036 (2012).

84. Korboukh, V. K., Li, N., Barr, E. W., Bollinger, J. M. Jr. & Krebs, C. A Long-Lived, Substrate-Hydroxylating Peroxodiiron(III/III) Intermediate in the Amine Oxygenase, AurF, from Streptomyces thioluteus. *J. Am. Chem. Soc.* **131**, 13608–13609 (2009).

85. Li, N., Korboukh, V. K., Krebs, C. & Bollinger, J. M. Four-electron oxidation of p-hydroxylaminobenzoate to p-nitrobenzoate by a peroxodiferric complex in AurF from Streptomyces thioluteus. *Proc. Natl. Acad. Sci.* **107**, 15722–15727 (2010).
