# Supplementary material for: Chemical capture of diazo metabolites reveals biosynthetic hydrazone oxidation
Source: Nature. 2026 Feb 4;652(8109):517–25. doi: 10.1038/s41586-025-10079-x (PMC13061610; doi:10.1038/s41586-025-10079-x)
Supplement: Supplementary file 2 — Reporting Summary [file 41586_2025_10079_MOESM2_ESM.pdf]

nature portfolio

Corresponding author(s): Emily Balskus

Last updated by author(s): Dec 14, 2025

Reporting Summary

Nature Portfolio wishes to improve the reproducibility of the work that we publish. This form provides structure for consistency and transparency in reporting. For further information on Nature Portfolio policies, see our Editorial Policies and the Editorial Policy Checklist.

Statistics

For all statistical analyses, confirm that the following items are present in the figure legend, table legend, main text, or Methods section.

n/a	Confirmed	
☐	☒	The exact sample size (*n*) for each experimental group/condition, given as a discrete number and unit of measurement
☐	☒	A statement on whether measurements were taken from distinct samples or whether the same sample was measured repeatedly
☐	☒	The statistical test(s) used AND whether they are one- or two-sided<br>*Only common tests should be described solely by name; describe more complex techniques in the Methods section.*
☒	☐	A description of all covariates tested
☒	☐	A description of any assumptions or corrections, such as tests of normality and adjustment for multiple comparisons
☐	☒	A full description of the statistical parameters including central tendency (e.g. means) or other basic estimates (e.g. regression coefficient) AND variation (e.g. standard deviation) or associated estimates of uncertainty (e.g. confidence intervals)
☐	☒	For null hypothesis testing, the test statistic (e.g. *F*, *t*, *r*) with confidence intervals, effect sizes, degrees of freedom and *P* value noted<br>*Give P values as exact values whenever suitable.*
☒	☐	For Bayesian analysis, information on the choice of priors and Markov chain Monte Carlo settings
☒	☐	For hierarchical and complex designs, identification of the appropriate level for tests and full reporting of outcomes
☒	☐	Estimates of effect sizes (e.g. Cohen's *d*, Pearson's *r*), indicating how they were calculated

Our web collection on statistics for biologists contains articles on many of the points above.

Software and code

Policy information about availability of computer code

Data collection	Data collection was performed using the following commercially available software: Thermo Xcalibur 4.5 was used for analysis using the Thermo Orbitrap IQ-X. Agilent MassHunter Workstation Data Acquisition Version 10.1 was used for analysis performed using the QTOF. Agilent ICP-MS MassHunter 4.5 version C.01.05 was used for ICP-MS analysis as well as ThermoFisher QTEGRA version 2.10 (2.10.4345.64) software. Agilent Carey UV Workstation 1.3.4 was used for UV-vis analysis. Cytiva UNICORN 7.7 was used for fast protein liquid chromatography and size exclusion chromatography. Azure biosystems version 1.8.0.1230 was used for gel imaging. Bruker TopSpin 4.4.0 was used to acquire NMR spectra.
Data analysis	Data analysis was performed using the following commercially available software: Compound Discoverer 3.3.1.111 was used for comparative metabolomics analysis. Prism 10.1.1 was used for data visualization. FreeStyle 1.8 SP2 version 1.8.63.0 was used for extracted ion chromatograph and MS/MS spectra analysis. Microsoft Excel Version 16.80 was used for calculations. PyMOL Molecular Graphics System version 3.0 was used for protein structure analysis. Agilent MassHunter Workstation Qualitative Analysis version 10.0 was used to analyze extracted ion chromatograms and MS/MS spectra. Geneious 2023.2.1 was used for bioinformatic analysis. JalView 2.11.4.1 was used for multiple sequence alignment visualization. Enzyme Function Initiative Genome Neighborhood Tool (EFI-GNT; 2019 release) was used for genomic neighborhood analysis. Cytoscape 3.10.1 was used for genome neighborhood network visualization.<br>MeRestNova 15.0.0-34764 was used to analyze NMR spectra. RStudio version 2023.12.0+369 was used to run prettyClusters analysis.<br>Data analysis was performed using prettyClusters tools, available on GitHub (https://github.com/g-e-kenney/prettyClusters)

For manuscripts utilizing custom algorithms or software that are central to the research but not yet described in published literature, software must be made available to editors and reviewers. We strongly encourage code deposition in a community repository (e.g. GitHub). See the Nature Portfolio guidelines for submitting code & software for further information.

Data

Policy information about availability of data

All manuscripts must include a data availability statement. This statement should provide the following information, where applicable:

- Accession codes, unique identifiers, or web links for publicly available datasets
- A description of any restrictions on data availability
- For clinical datasets or third party data, please ensure that the statement adheres to our policy

Genome mining was performed using the NCBI reference protein database. Raw LC–MS data and LC–MS/MS data are available upon request due to the large file size. Previously published crystal structures are available in the Protein DataBank (https://www.rcsb.org/) under accession codes 3CHH and 5HYH. All other data are available in the manuscript or Supplementary Information. Source data are provided with this paper.

Research involving human participants, their data, or biological material

Policy information about studies with human participants or human data. See also policy information about sex, gender (identity/presentation), and sexual orientation and race, ethnicity and racism.

Reporting on sex and gender	Humans were not studied and this information was therefore not collected.
Reporting on race, ethnicity, or other socially relevant groupings	Humans were not studied and this information was therefore not collected.
Population characteristics	Humans were not studied and this information was therefore not collected.
Recruitment	Humans were not studied and this information was therefore not collected.
Ethics oversight	Humans were not studied and this information was therefore not collected.

Note that full information on the approval of the study protocol must also be provided in the manuscript.

Field-specific reporting

Please select the one below that is the best fit for your research. If you are not sure, read the appropriate sections before making your selection.

☒ Life sciences ☐ Behavioural & social sciences ☐ Ecological, evolutionary & environmental sciences

For a reference copy of the document with all sections, see nature.com/documents/nr-reporting-summary-flat.pdf

Life sciences study design

All studies must disclose on these points even when the disclosure is negative.

Sample size	*Describe how sample size was determined, detailing any statistical methods used to predetermine sample size OR if no sample-size calculation was performed, describe how sample sizes were chosen and provide a rationale for why these sample sizes are sufficient.*
Data exclusions	No data was excluded from analysis.
Replication	Experiments were performed in biological triplicates to ensure reproducibility, except for ICP-MS experiments which were performed in biological duplicates due to material limitations. All attempts at replication were successful.
Randomization	Randomization was not required as there were no experimental groups.
Blinding	Blinding was not relevant as no group allocation was performed.

Reporting for specific materials, systems and methods

We require information from authors about some types of materials, experimental systems and methods used in many studies. Here, indicate whether each material, system or method listed is relevant to your study. If you are not sure if a list item applies to your research, read the appropriate section before selecting a response.

Materials & experimental systems

n/a	Involved in the study
☒	☐ Antibodies
☒	☐ Eukaryotic cell lines
☒	☐ Palaeontology and archaeology
☐	☒ Animals and other organisms
☒	☐ Clinical data
☒	☐ Dual use research of concern
☒	☐ Plants

Methods

n/a	Involved in the study
☒	☐ ChIP-seq
☒	☐ Flow cytometry
☒	☐ MRI-based neuroimaging

Animals and other research organisms

Policy information about studies involving animals; ARRIVE guidelines recommended for reporting animal research, and Sex and Gender in Research

Laboratory animals	The study did not involve laboratory animals.
Wild animals	The study did not involve wild animals.
Reporting on sex	As the study did not involve animals, sex was not reported on.
Field-collected samples	The study did not include samples collected from the field.
Ethics oversight	No ethical guidance was required as the work was performed with bacterial strains.

Note that full information on the approval of the study protocol must also be provided in the manuscript.

Plants

Seed stocks	Seed stocks were not used.
Novel plant genotypes	No novel plant genotypes were produced.
Authentication	No plants were used and therefore no authentication procedures were used.
